# Development of crow search algorithm using the characteristics of qubits and application of engineering problems

**DOI:** 10.1038/s41598-025-30625-x

**Published:** 2026-01-03

**Authors:** Donwoo Lee, Seungjae Lee

**Affiliations:** School of Industrial Design & Architectural Engineering, Korea University of Technology & Education, 1600 Chungjeol-ro, Byeongcheon-myeon, Cheonan, 31253 Chungcheongnam-do Republic of Korea

**Keywords:** Quantum computing, Qubit, Meta-heuristics, Crow search algorithm, Engineering problem, Engineering, Mathematics and computing, Physics

## Abstract

Recently, researchers have attempted to develop a new algorithm by combining quantum systems and metaheuristics algorithms and are confirming its applicability in engineering optimization problems. This paper proposes a new QbCSA (quantum-based crow search algorithm) combining quantum systems and CSA (crow search algorithm). Unlike CSA, the initial matrix of QbCSA consists of qubits and performs operations through spin and measurement processes. Six benchmark functions were used to compare the convergence performance according to the parameter change used in the developed QbCSA, and the optimal parameter range is suggested. In addition, the CEC2019 benchmark functions and four engineering example problems were solved and compared with the results of previous studies. QbCSA demonstrated comparable performance to CSA, which uses decimal-based design variables, while achieving lower variance and more stable convergence than QbHSA. In particular, for multimodal optimization problems, QbCSA exhibited superior search efficiency and solution diversity. Furthermore, the four engineering examples confirmed the practical applicability of QbCSA, and these results indicate that qubit-based encoding can enhance the search efficiency of CSA and suggest broader applicability to engineering optimization problems.

## Introduction

 The development of computers has played many roles as a tool to solve various engineering problems for engineers. However, the development of quantum computers is accelerating to solve the physical limitations of computers that cannot be smaller and more complex engineering problems. The development of quantum computers has many steps to go to the commercialization stage, but Google, IBM (International Business Machines Corporation), and KRISS (Korea Research Institute of Standards and Science) have already verified that their computation speed is faster than that of existing computers^1^. It is evident that the development of technology in quantum computing, using quantum mechanics and a physical device called a quantum computer, is attracting the attention of many engineers^2,3^.

The most significant difference between quantum computing and existing computers is the minimum information processing unit for processing operations. Quantum computing uses qubits while existing computers use bits. The bit of an existing computer only exists in a state of 0 or 1, but the qubit exists in a state in which 0 and 1 are superimposed as probabilities, and this state is expressed as the superposition of the two^4^. Therefore, qubits can express much more information simultaneously than bits, and due to these characteristics, operations can be performed faster^5^. Feynman first established quantum systems in 1982^6^, and Deutsch demonstrated that data processing was possible using quantum systems in 1983^7^. Since then, many attempts have been made to realize quantum systems and solve problems efficiently^8,9^. In particular, Shor’s prime factorization algorithm in 1994 and Grover’s search data search algorithm in 1996 began to attract the attention of many researchers^10,11^ and are being developed to solve optimization problems in aerospace, chemistry, logistics, and robotics. Despite the growing body of research on quantum-inspired metaheuristics, systematic investigations into how quantum-specific parameters influence algorithmic convergence remain scarce^5^. Existing studies have primarily focused on demonstrating the potential of quantum-based representations, but few have examined the detailed parameter dynamics that govern convergence stability and solution quality. The authors address this gap by conducting a comprehensive statistical analysis of the quantum-based metaheuristics algorithm’s key parameters, including qubit count, measurement frequency, and rotation angle, across multiple benchmark functions. This approach not only quantifies the performance impact of each parameter but also establishes recommended ranges that balance convergence speed and solution accuracy. By bridging quantum-inspired algorithm design with rigorous parameter tuning, the present study contributes a reproducible methodology for enhancing the performance of quantum-based metaheuristics, which can be extended to other algorithmic frameworks.

This paper aims to propose QbCSA (quantum-based crow search algorithm) by combining quantum systems and CSA (crow search algorithm) among metaheuristic algorithms and to identify QbCSA’s convergence performance to confirm its possibility of solving engineering problems. Recently, the tendency to study metaheuristic algorithms has increased significantly due to their advantage of solving NP-hard problems in polynomial time^12,13^. Metaheuristic algorithms are classified into various algorithms according to their motif evolution, swarm, physics, and human behavior^14^. Due to the ample domain space, they can solve problems that are difficult to explore with traditional methods^15^. In 2000, Han and Kim proposed GQA (genetic quantum algorithm) and were the first to calculate GA (genetic algorithm) using the concept and superposition state of the qubit^16^. They compared the convergence performance of GQA and GA using the knapsack problem and confirmed that the global search ability was superior to the qubit’s probabilistic data. Based on their research, a new field is being created by combining various metaheuristic algorithms whose efficiency has been verified. In 2021, Sadeghi Hesar et al. proposed QMOHSA (quantum multi-objective harmonic search algorithm), and analysis and task scheduling problems according to parameters were performed^17^. In 2024, Zhu et al. proposed a double bead search algorithm on quantum computing and a multi-state hybrid (QHDBO), confirming that convergence performance is superior to conventional metaheuristics algorithms^18^. In addition, it is constantly being developed in metaheuristics algorithms such as PSO (particle swarm optimization) and GWO (gray wolf optimizer) based on quantum systems^19–21^. This quantum-based approach has been reported in several studies to enhance global search capability through probability amplitude–based exploration and to be effective in preventing premature convergence. For example, Lee et al. proposed a quantum computing–based harmony search algorithm and applied it to weight optimization of truss structures, verifying that the use of quantum computing is meaningful for solving architectural optimization problems^22^. Fahad et al. (2023) proposed a quantum-inspired particle swarm optimization hybrid model, and demonstrated that it achieved improvements in both convergence speed and solution quality compared to conventional PSO in solving multimodal electromagnetic design problems^23^. These results suggest that optimization techniques incorporating quantum concepts can exhibit superior performance in large-scale and complex design spaces^24^. Therefore, incorporating qubit-based representation into metaheuristic algorithms has great potential to simultaneously improve search efficiency and solution accuracy, and future studies should verify its effectiveness across various algorithms.

The metaheuristic algorithm adopted by the authors to incorporate quantum systems is CSA which was proposed by Askarzadeh in 2016^25^. CSA has been proposed to describe the behavior of crows using high intelligence to chase food. Its simple application and computation are applied to various fields, such as energy problems, optimal design of structures, and optimal control problems^26–29^. In addition, many researchers attempt to improve convergence performance by proposing dynamic parameters that change with generational numbers using fixed values or by adding new mathematical expressions or other metaheuristic algorithms. In 2019, Pamir et al. proposed a BCSA (bat crow search algorithm) that combines BA (bat algorithm) and CSA and confirmed that the convergence performance was improved through the solution of smart grid applications^30^. In 2020, Wu et al. proposed LFCSA (crow search algorithm with Levi flight) with the added Levi flight formula^31^. They confirmed that global search capabilities have improved through FEMU (finite element model updating). In 2022, Necira et al. proposed a DCSA (dynamic crow search algorithm) using parameters that dynamically change according to the number of generations and confirmed that convergence performance was improved over CSA using benchmark functions^32^. In 2023, Lee et al. proposed an ACSA (advanced crow search algorithm) using dynamic parameters and equations to combine exploitation and exploration properly^14^. They confirmed that convergence performance was improved over other metaheuristic algorithms through benchmark function and engineering problem-solving. There needs to be more cases in which CSA, which is being improved in various directions as described above, is proposed to be operated using a quantum system. However, it is required to respond to future technologies by confirming in advance that optimization using metaheuristics algorithms can be applied to the field of quantum computers and suggesting various measures to improve convergence performance.

Therefore, this paper implements QbCSA, calculated using a quantum system using qubit, as MATLAB R2023a. We compare the convergence performance that changes according to the parameter change of QbCSA using six benchmark functions and propose an optimal parameter range. In addition, it is confirmed that practical engineering problems can be solved by applying QbCSA to four engineering problems. The composition of the paper is as follows: Sect. 2 describes the calculation process of QbCSA, Sect. 3 compares the convergence performance according to parameter changes, Sect. 4 solves six benchmark functions and four engineering problems, and compares the results with other algorithms. The final Sect. 5 concludes.

## Quantum based crow search algorithm

### Qubit status and operator

**Fig. 1:**
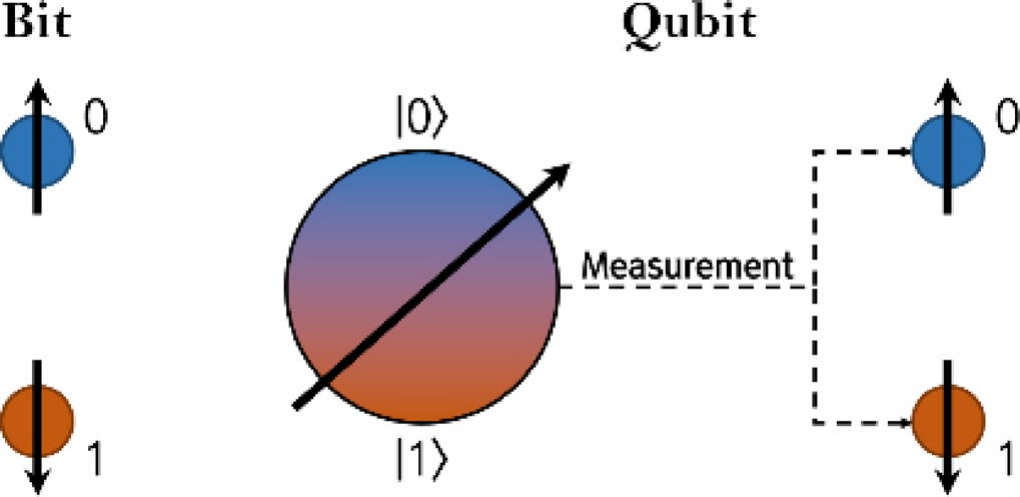
Bit and qubit state.

Figure 1 is a diagram representing the states of bit and qubit. As the introduction mentions, qubit exists where 0 and 1 superposition and is expressed as a value of 0 or 1 through measurement. The basic state of a single qubit can be expressed as Eq. (1), and $\:\alpha\:$ and $\:\beta\:$ are the probability amplitudes of $\left| 0 \right\rangle$ and $\left| 1 \right\rangle$^33^. Equation (2) is established for the probability of a state derived after measurement in a superposition state according to the Born rule of the absolute value of the probability amplitude^34^. In addition, if the state of a single qubit is expressed as a vector, it can be expressed as Eq. (3).

1 $$\left| \psi \right\rangle = \alpha \left| 0 \right\rangle + \beta \left| 1 \right\rangle$$

2 $$\:{\left|\alpha\:\right|}^{2}+{\left|\beta\:\right|}^{2}=1$$

3 $$\:q=\:\left[\begin{array}{c}\alpha\:\\\:\beta\:\end{array}\right]$$

**Fig. 2:**
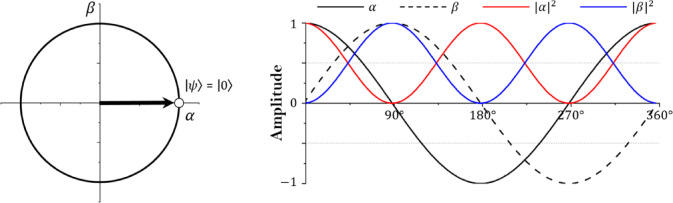
Single qubit state.

Figure 2 shows a single qubit in a two-dimensional vector state and the amplitude change according to the qubit’s state space converging to 0. The state space of the single qubit is in the form of a circle, and $\:\alpha\:$ and $\:\beta\:$ are complex numbers. $\:\alpha\:$ and $\:\beta\:$ are in an orthogonal state and may have values from − 1 to 1^35^. In addition, the absolute value square root of each probability amplitude has a value from 0 to 1, and the sum of $\:{\left|\alpha\:\right|}^{2}$ and $\:{\left|\beta\:\right|}^{2}$ according to Eq. 2 always satisfies 1. If multiple qubits exist, the state of m square bits is defined as Eq. (4).

4 $$\:\varvec{q}=\left[{q}_{1}\:{q}_{2}\:\cdots\:\:{q}_{m-1}\:{q}_{m}\right]=\left[\begin{array}{c}{\alpha\:}_{1}\\\:{\beta\:}_{1}\end{array}\:\begin{array}{c}{\alpha\:}_{2}\\\:{\beta\:}_{2}\end{array}\:\begin{array}{c}\cdots\:\\\:\cdots\:\end{array}\:\begin{array}{c}{\alpha\:}_{m-1}\\\:{\beta\:}_{m-1}\end{array}\:\begin{array}{c}{\alpha\:}_{m}\\\:{\beta\:}_{m}\end{array}\right]$$

**Fig. 3:**
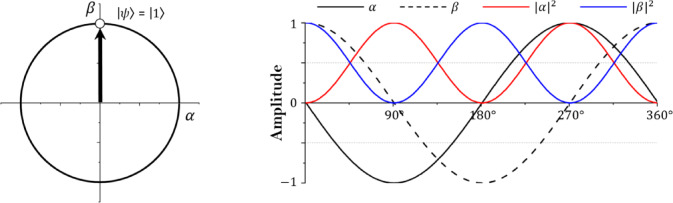
Single qubit state through Pauli gate.

**Fig. 4:**
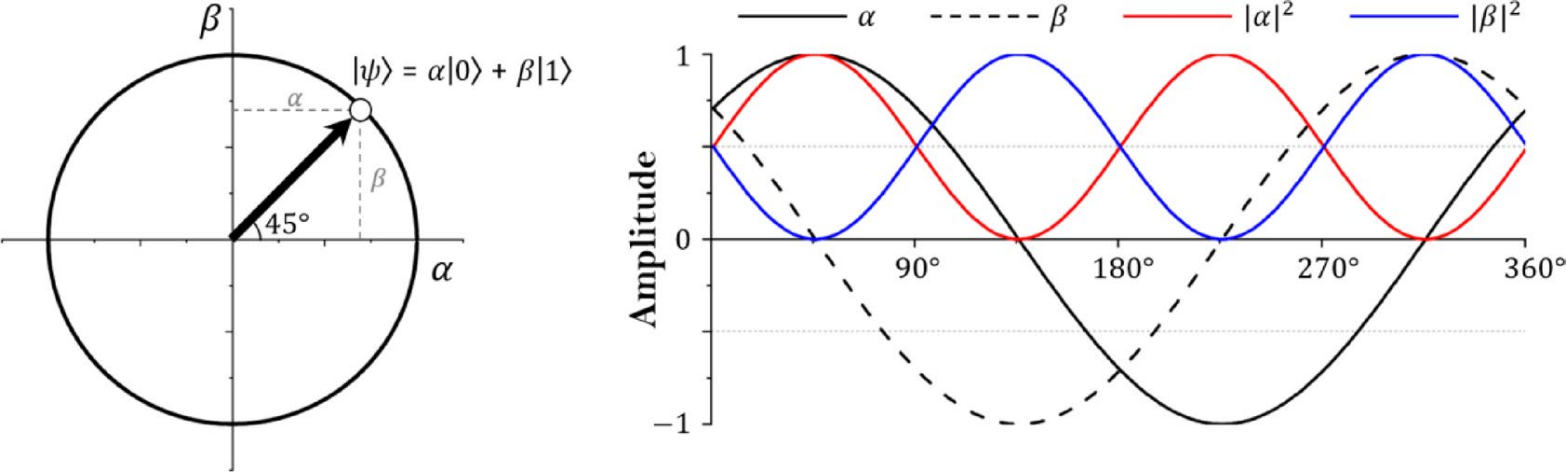
Single qubit state through Hadamard gate.

In a gate-based quantum system, quantum operators that change the state of qubits are unitary and have reversible characteristics^36^. Representative operators are Pauli gate, Hadamard gate, H_ε_ gate, and Rotation gate. These quantum operators are classified as a single quantum operator and are represented by a 2 × 2 matrix^37^. Among them, the Pauli gate and Hadamard gate are defined by Eqs. (5) and (6), respectively. The Pauli gate plays a role similar to the NOT gate in classical computing, swapping the amplitudes of $\left| 0 \right\rangle$ and $\left| 1 \right\rangle$. The Hadamard gate transforms a qubit state that has fully collapsed to 0 or 1 into a linear superposition state. Notably, the Hadamard gate has the self-inverse property and is considered one of the most important quantum operators because it generates superposition states. When a single qubit in the initial state shown in Fig. 1 passes through each gate, its state changes as illustrated in Figs. 3 and 4.

5 $$\:\widehat{P}=\left[\begin{array}{cc}0&\:1\\\:1&\:0\end{array}\right]$$

6 $$\:\widehat{H}=\frac{1}{\sqrt{2}}\left[\begin{array}{cc}1&\:1\\\:1&\:-1\end{array}\right]$$

**Fig. 5:**
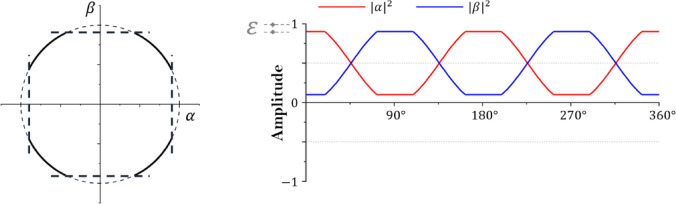
Concept of H_ε_ gate.

H_ε_ gate is one of the quantum operators first proposed by Han and Kim in 2004^38^. It is used to prevent the complete convergence of qubits to 0 or 1. H_ε_ gate is classified and calculated as Eq. (7) according to the positions of $\:\alpha\:$ and $\:\beta\:$ in the qubit. This role helps escape from the local minima when the qubit converges to 0 or 1. The concept of H_ε_ gate can be expressed as in Fig. 5.

7 $$if~\left| \alpha \right|^{2} \le \varepsilon ~and~\left| \beta \right|^{2} \ge 1 - \varepsilon \, \to \,\left[ {\begin{array}{*{20}c} \alpha \\ \beta \\ \end{array} } \right] = \left[ {\begin{array}{*{20}c} {\sqrt \varepsilon } \\ {\sqrt {1 - \varepsilon } } \\ \end{array} } \right]$$

elseif $$\:{\left|\alpha\:\right|}^{2}\ge\:1-\epsilon\:$$ and $$\:{\left|\beta\:\right|}^{2}\le\:\epsilon\:$$ → $$\:\left[\begin{array}{c}\alpha\:\\\:\beta\:\end{array}\right]=\left[\begin{array}{c}\sqrt{1-\epsilon\:}\\\:\sqrt{\epsilon\:}\end{array}\right]$$.

otherwise → $$\:\left[\begin{array}{c}\alpha\:\\\:\beta\:\end{array}\right]=\left[\begin{array}{c}\alpha\:\\\:\beta\:\end{array}\right]$$.

Finally, the rotation gate is a quantum operator that performs qubit rotation concerning the current generation based on the previous generation. The rotation gate is defined as an Eq. (8), and *t* and $\:\theta\:$ represent the current number of generations and the rotation angle of the qubit^39^. The qubit’s rotation angle can be expressed as Eq. (9) and $\:\varDelta\:\theta\:$ is determined by Table 1. $\:{x}_{i}$ and $\:{b}_{i}$ in Table 1 represent the current generation binary string of the i-th qubit and the previous generation optimal binary string.

8 $$\:\left\{\begin{array}{c}{\alpha\:}^{t+1}\\\:{\beta\:}^{t+1}\end{array}\right\}=\left[\begin{array}{cc}\text{c}\text{o}\text{s}\left(\theta\:\right)&\:-\text{s}\text{i}\text{n}\left(\theta\:\right)\\\:\text{s}\text{i}\text{n}\left(\theta\:\right)&\:\text{c}\text{o}\text{s}\left(\theta\:\right)\end{array}\right]\left\{\begin{array}{c}{\alpha\:}^{t}\\\:{\beta\:}^{t}\end{array}\right\}$$

9 $$\:\theta\:=\varDelta\:\theta\:\times\:sign\left({\alpha\:}_{i}{\beta\:}_{i}\right)$$

**Table 1:** Lookup table for qubit rotation.

$\:{x}_{i}$	$\:{b}_{i}$	$\:f\left(x\right)<f\left(b\right)$	$\:\varDelta\:\theta\:$	$\:sign\left({\alpha\:}_{i}{\beta\:}_{i}\right)$			
				$\:{\alpha\:}_{i}{\beta\:}_{i}>0$	$\:{\alpha\:}_{i}{\beta\:}_{i}<0$	$\:{\alpha\:}_{i}=0$	$\:{\beta\:}_{i}=0$
0	0	True	0	0	0	0	0
0	0	False	0	0	0	0	0
0	1	True	$\:{\theta\:}_{p}$	1	-1	0	± 1
0	1	False	0	0	0	0	0
1	0	True	$\:{-\theta\:}_{p}$	1	-1	± 1	0
1	0	False	0	0	0	0	0
1	1	True	0	0	0	0	0
1	1	False	0	0	0	0	0

* $$\:{\theta\:}_{p}={\theta\:}_{r}\times\:\pi\:$$.

### Procedure of QbCSA

Like CSA, QbCSA performs optimization in 8 steps, but the two algorithms differ in configuring design variables. The design variables of the CAS are composed of decimal numbers, and the decimal design variables are directly adjusted according to the proposed equation. The design variables of QbCSA are composed of qubits, and superposition probabilities are composed of design variables. In addition, the proposed equation adjusts the superposition probability, and the quantum operators change the qubit state. The measured results can confirm the qubit’s state, and this feature makes it difficult to compare it with a general meta-heuristic algorithm using decimals directly.

The procedure of QbCSA is as follows.

Step 1: Set the parameters

Step 1 defines the optimization problem and defines the parameters. Like CSA, N (flock size), *t*_max_ (maximum generation), fl. (flight length), and AP (awareness probability) are defined, and the use of quantum systems adds new parameters. The added parameters are nQ (number of qubits), nM (number of measurements), $\:\epsilon\:$, and $\:{\theta\:}_{R}$.

Step 2: Initialize position and memory of crows

In Step 2, the initial position of the crow is determined, as shown in Eq. (10). Since CSA uses decimal, each design variable is composed of random decimal numbers within the scope of the problem. However, since each design variable of QbCSA uses qubit, it is composed in the same form as Eq. (4). The initial probability of QbCSA initializes the probability that 0 or 1 is selected to the same probability and passes through the H_ε_ gate. The initial position of the crow is remembered by the crow and used later for the evaluation and comparison of the crow position of the current generation and adjusted generations’ crow positions.

**Fig. 6:**
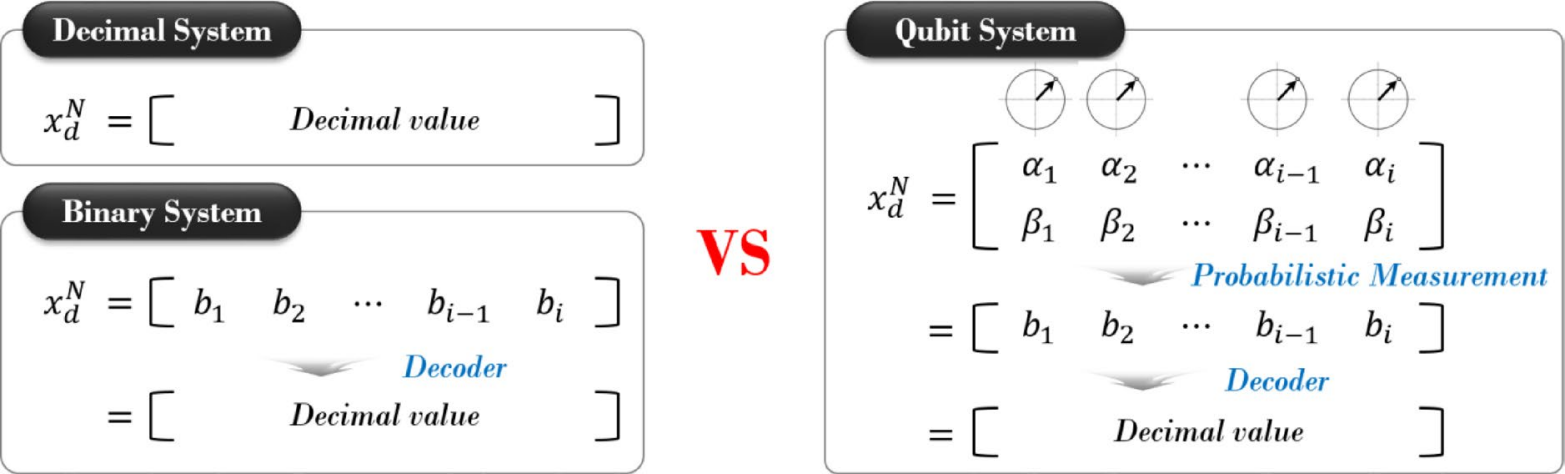
Comparison of design variable representation in decimal, binary, and qubit systems.

Figure 6 illustrates the conceptual difference between decimal-, binary-, and qubit-based representations. The decimal system directly encodes the variable as a real number, the binary system encodes it as a bit string and decodes it into a real value, whereas the qubit system utilizes probabilistic measurement of quantum states before decoding into the design variable. This qubit representation enables probabilistic exploration of the solution space, potentially enhancing global search capability and avoiding premature convergence.

10 $$Crows = \left[ {\begin{array}{*{20}c} {x_{1}^{1} } & \cdots & {x_{d}^{1} } \\ \vdots & \ddots & \vdots \\ {x_{1}^{N} } & \cdots & {x_{d}^{N} } \\ \end{array} } \right]$$

Step 3: Evaluate fitness function

In Step 3, the position of the crow initialized in Step 2 is evaluated. CSA directly evaluates using the design variables of decimal. In QbCSA, the qubit measurement and the decoding of converting the binary number derived through the measurement to decimal are added. If the number of measurements is two or more, measure qubit by the number of measurements and remember better results.

Step 4: Generate new position

**Fig. 7:**
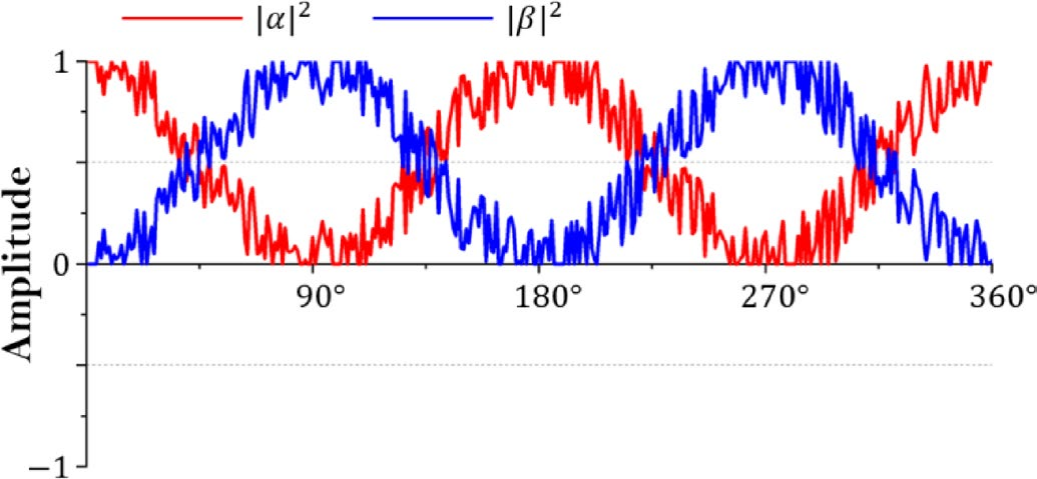
Adjustment effect in QbCSA.

Step 4 is the most critical step in performing optimization and adjusting the position of the crow. The position of the crow in the CSA is adjusted by Eq. (11). If the random number (*r*) is greater than or equal to the AP, the crow of the current generation ($\:{x}^{t}$) follows the randomly selected crow ($\:{m}^{t}$). Otherwise, the crow of the current number of households moves randomly within the scope of the problem. QbCSA shows the effect shown in Fig. 7 by adjusting the probability amplitude value of qubit by Eq. (11) or passing through the Hadamard gate to superposition the qubit state. After that, the adjusted probability amplitude of the qubit passes through the H_ε_ gate to suppress the convergence of the qubit.

11 $$\:{x}^{t+1}=\left\{\begin{array}{c}{x}^{t}+r\times\:fl\times\:\left({m}^{t}-{x}^{t}\right)\\\:random\:position\end{array}\right.\:\begin{array}{c}if\:r\ge\:AP\\\:otherwise\end{array}$$

In addition, these adjustments generally have a different computational process than meta-heuristics algorithms using decimal. Therefore, it is not easy to directly compare quantum-based meta-heuristics algorithms with general metaheuristics algorithms.

Step 5: Check the feasibility of new positions

In Step 5, the CSA checks if the crow’s location is within the problem’s boundary and does not adjust if it is not. QbCSA uses the probability amplitude of the qubit.

Step 6: Evaluate fitness function

In Step 6, evaluate the location of the new crow adjusted in Step 4. As in Step 3, measurement and decoding are performed.

Step 7: Update memory

In Step 7, better results are updated in crow memory by comparing the evaluation of existing crow locations with the evaluation of adjusted crow locations.

Step 8: Check termination criterion

QbCSA performs a repetitive operation in Steps 4–7 until *t* reaches *t*_max_ and shows the optimization result when *t*_max_ is reached. The meta-heuristics algorithm to which the quantum system is applied can use the probability average of qubits as well as the number of generations as a termination condition. Equations (12) and (13) define the termination condition by the probability average of the qubit, and the algorithm terminates faster as the magnitude of $\:{\upgamma\:}$ increases^38^. The new termination condition suggests the possibility of deriving a result faster and of deriving an optimal result only by the measurement process of the qubit.

12 $$\:{C}_{b}\left(\varvec{q}\right)=\frac{1}{m}\sum\:_{i=1}^{m}\left|1-2{\left|{\alpha\:}_{i}\right|}^{2}\right|\:\text{o}\text{r}\:{C}_{b}\left(\varvec{q}\right)=\frac{1}{m}\sum\:_{i=1}^{m}\left|1-2{\left|{\beta\:}_{i}\right|}^{2}\right|$$

13 $$\:{C}_{av}=\left(\frac{1}{n}\sum\:_{j=1}^{n}{C}_{b}\left({\varvec{q}}_{j}\right)\right)>\gamma\:$$

## Analysis of convergence performance according to parameter changes

This study aims to determine the optimal parameter ranges of QbCSA by comparing convergence performance according to changes in parameter values. For the comparison, a total of six benchmark functions were used, as shown in Table 2. Among these, f1–f4 evaluate the exploitation capability of the algorithm, while f5–f6 assess the exploration capability. All simulations were run for 1,000 generations, and the comparison was based on the mean fitness values averaged over 20 independent runs. Since the resulting values vary significantly depending on the function, the results were normalized to the range [1, 1000], where values closer to 1 indicate better convergence performance. In addition, to statistically verify the significance of performance differences and the ranking trends according to parameter variations, both the Friedman test and Page’s L test were conducted. For both tests, the p-value indicates the probability that the observed differences or trends occurred by chance, and a value of *p* < 0.05 is considered statistically significant. The Friedman test evaluates whether there are significant differences in performance among parameter settings, while Page’s L test examines whether there is a consistent trend in performance ranking in a specific direction^40,41^.

**Table 2:** Benchmark function for parameters analysis.

Index	Name	Type	Boundary	Min
f1	Sphere function	Unimodal	[-100 100]	0
f2	Schwefel’s problem 2.22	Unimodal	[-10 10]	0
f3	Schwefel’s problem 1.2	Unimodal	[-100 100]	0
f4	Generalized Rosenbrock’s function	Unimodal	[-30 30]	0
f5	Ackley’s function	Multimodal	[-32 32]	0
f6	Generalized Griewank function	Multimodal	[-600 600]	0

### N (flock size)

N represents the population size and is an essential factor for determining the size of the initially determined crow position matrix. As the value of N increases, the size of the matrix increases; conversely, as the value of N decreases, the size of the matrix decreases. For the comparison of convergence performance, 1, 5, 10, 15, 20, 25, 30, 40, and 50 were selected as values of N, and other parameters were fixed as shown in Table 3.

**Table 3:** Parameters for N analysis.

System	Parameters
For CSA	Dimension of problem (d): 5 Awareness probability (AP): 0.1 Flight length (fl.): 2
For qubits	Number of qubits (nQ): 20 Number of measurements (nM): 1 $\:{\upepsilon\:}$ for H_ε_ gate: 0.1 Rotation angle ($\:{\theta\:}_{r}$): 0.06

**Fig. 8:**
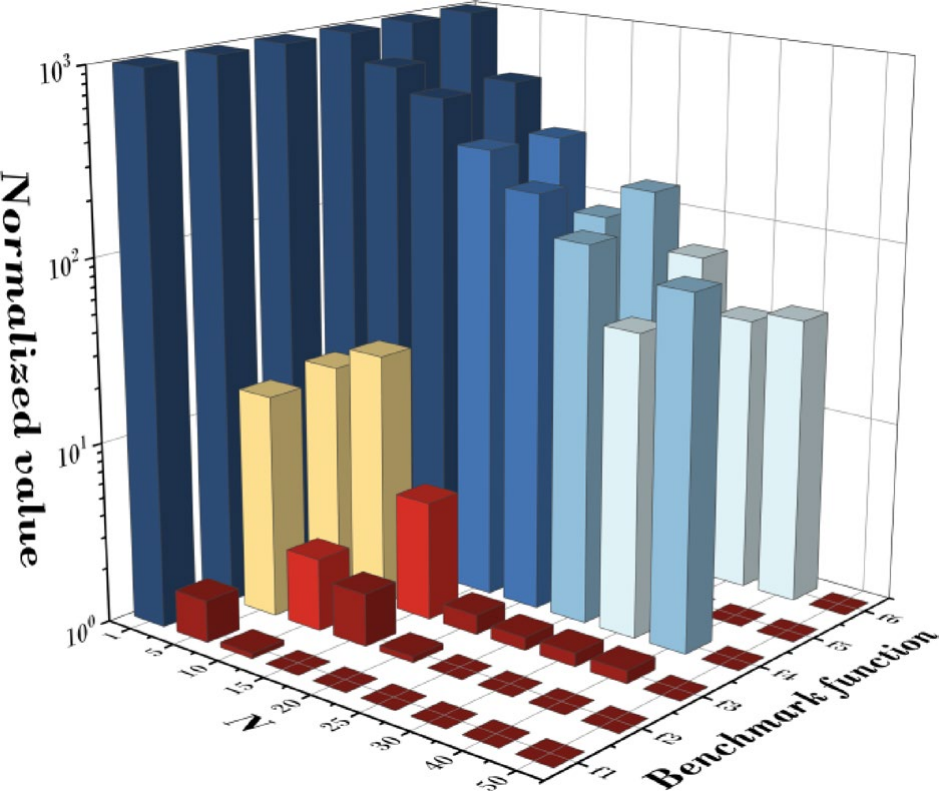
Results according to change of N.

Figure 8; Table 4 presents the results of statistical validation based on the mean fitness values obtained from 20 independent runs for six benchmark functions (F1–F6) at each N value. A Friedman value closer to 1 indicates superior convergence performance. The analysis shows that when *N* = 1, the convergence performance was the lowest for all functions, and as N increased, the performance improved progressively. In particular, at *N* = 50, all functions recorded excellent performance, yielding a Friedman value of 1.00. For statistical significance testing, the Friedman test was conducted, resulting in X^2^_F_ = 41.83, *p* = 6.39 × 10E − 8 (*p* < 0.001), confirming that the performance differences according to N are highly significant. Furthermore, the Page’s L test was performed to examine the monotonic trend of the rankings, yielding L = 1015.0, *p* = 0.00 (*p* < 0.001), which strongly supports the finding that performance improves as N increases. Specifically, in terms of mean fitness (M.F.), the values obtained when *N* = 50 were 4.547E-08 for f1, 4.768E-05 for f2, 7.295E-05 for f3, 3.639E + 01 for f4, 1.221E-04 for f5, and 3.803E-02 for f6. In all functions, the performance was the lowest at *N* = 1, and improved as N increased.

These results suggest that a larger N increases the exploration range of the population, providing more diverse search paths and thereby raising the probability of finding better solutions. Therefore, in QbCSA, setting N to as large a value as feasible is advantageous for achieving stable and superior convergence performance. For reference, Table A1 in the Appendix provides the detailed best fitness (B.F.), mean fitness (M.F.), and standard deviation (Std.) values for each function.

**Table 4:** Friedman value for change of N.

*N*	1	5	10	15	20	25	30	40	50
Friedman value	9.00	7.83	7.17	5.83	4.50	2.33	1.83	1.83	1.00
Friedman test	X^2^_F_ = 41.83, *p* = 6.39E-8								
Page’s test	L = 1015.00, *p* = 1.39E-19								

### nQ (number of qubits)

nQ means the number of qubits, and the range of the number of expressions varies depending on the size of nQ. That is, as nQ increases, the range of expressions increases; as nQ decreases, the range of expressions decreases. For the comparison of convergence performance, 1, 3, 5, 10, 15, 20, 30, 40, and 50 were selected as values of nQ, and other parameters were fixed as shown in Table 5.

**Table 5:** Parameters for nQ analysis.

System	Parameters
For CSA	Dimension of problem (d): 5 Flock size (N): 10 Awareness probability (AP): 0.1 Flight length (fl.): 2
For qubits	Number of measurements (nM): 1 $\:{\upepsilon\:}$ for H_ε_ gate: 0.1 Rotation angle ($\:{\theta\:}_{r}$): 0.06

**Fig. 9:**
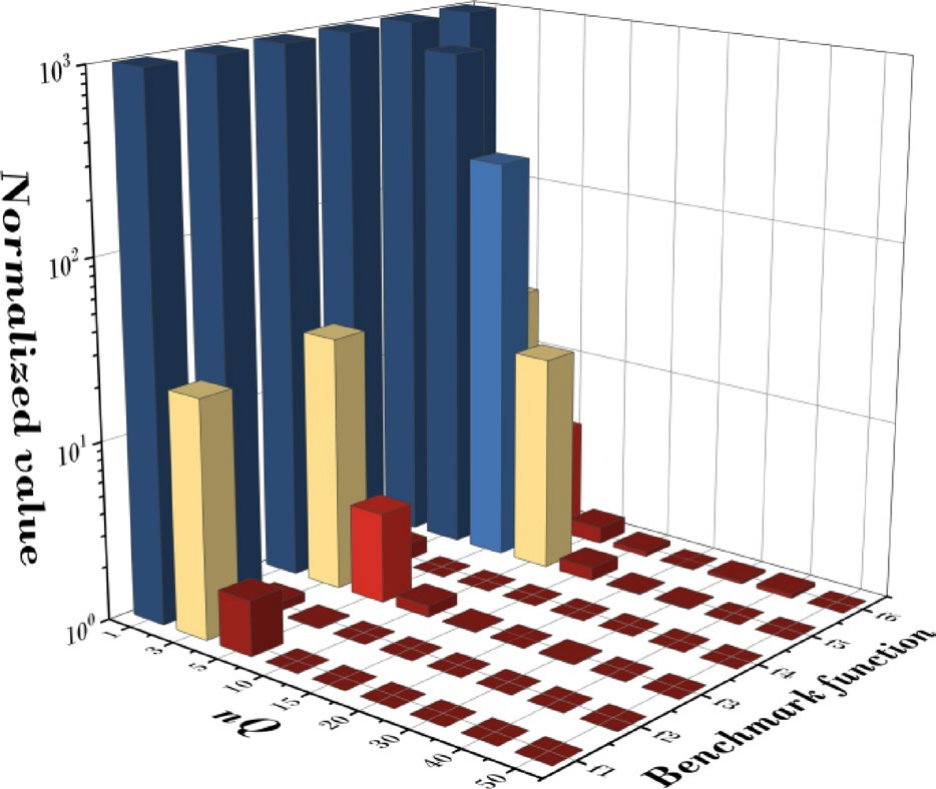
Results according to change of nQ.

Figure 9; Table 6 present the statistical validation results based on the mean fitness values obtained from 20 independent runs for six benchmark functions (f1–f6) at each nQ value. A smaller Friedman value indicates superior convergence performance. The analysis shows that when nQ = 1, the convergence performance was the lowest for all functions, and as nQ increased, the performance generally improved. In particular, notable improvements were observed up to nQ = 30, after which the performance gains tended to level off. This suggests that once the expression range is sufficiently large, further increases in nQ have a reduced impact on convergence. For statistical significance testing, the Friedman test was conducted, resulting in X^2^_F_ = 40.22, *p* = 1.79E-7 (*p* < 0.001), confirming that the performance differences according to nQ are highly significant. Furthermore, the Page’s L test was performed to examine the monotonic trend of the rankings, yielding L = 984.0, *p* = 0.00 (*p* < 0.001), which strongly supports the finding that performance improves as nQ increases. Specifically, in terms of mean fitness (M.F.), the best results for each function were 1.259E-07 for f1 at nQ = 30, 4.495E-05 for f2 at nQ = 40, 1.500E-01 for f3 at nQ = 50, 3.213E + 01 for f4 at nQ = 30, 1.873E-04 for f5 at nQ = 40, and 6.101E-02 for f6 at nQ = 50.

These results indicate that a larger nQ value expands the search space, providing more diverse exploration paths and increasing the likelihood of finding better solutions. However, since performance improvements diminish beyond nQ = 30, selecting nQ within a range that balances accuracy and computational cost may be optimal. For reference, Table A2 in the Appendix provides the detailed best fitness (B.F.), mean fitness (M.F.), and standard deviation (Std.) values for each function.

**Table 6:** Friedman value for change of nQ.

nQ	1	3	5	10	15	20	30	40	50
Friedman value	9.00	8.00	7.00	6.00	4.50	3.50	2.67	2.33	2.00
Friedman test	X^2^_F_ = 40.22, *p* = 1.79E-7								
Page’s test	L = 984.00, *p* = 0.00								

### nM (number of measurement)

nM means the number of times a qubit is measured, and a qubit expressed as the probability is expressed in binary through the measurement process. As the number of measurements increases by the qubit expressed as a probability, a better value is likely to be derived. For the convergence performance comparison, nM values were selected as 1, 2, 4, 6, 8, and 10, and other parameters were fixed as shown in Table 7. In particular, if the number of households is large, some convergence results may be derived from all results. Therefore, *t*_max_ is set short to 100 and compared according to the measurement of the qubit.

**Table 7:** Parameters for nM analysis.

System	Parameters
For CSA	Maximum generation (*t*_max_): 100 Dimension of problem (d): 5 Flock size (N): 10 Awareness probability (AP): 0.1 Flight length (fl.): 2
For qubits	Number of qubits (nQ): 20 $\:{\upepsilon\:}$ for H_ε_ gate: 0.1 Rotation angle ($\:{\theta\:}_{r}$): 0.06

**Fig. 10:**
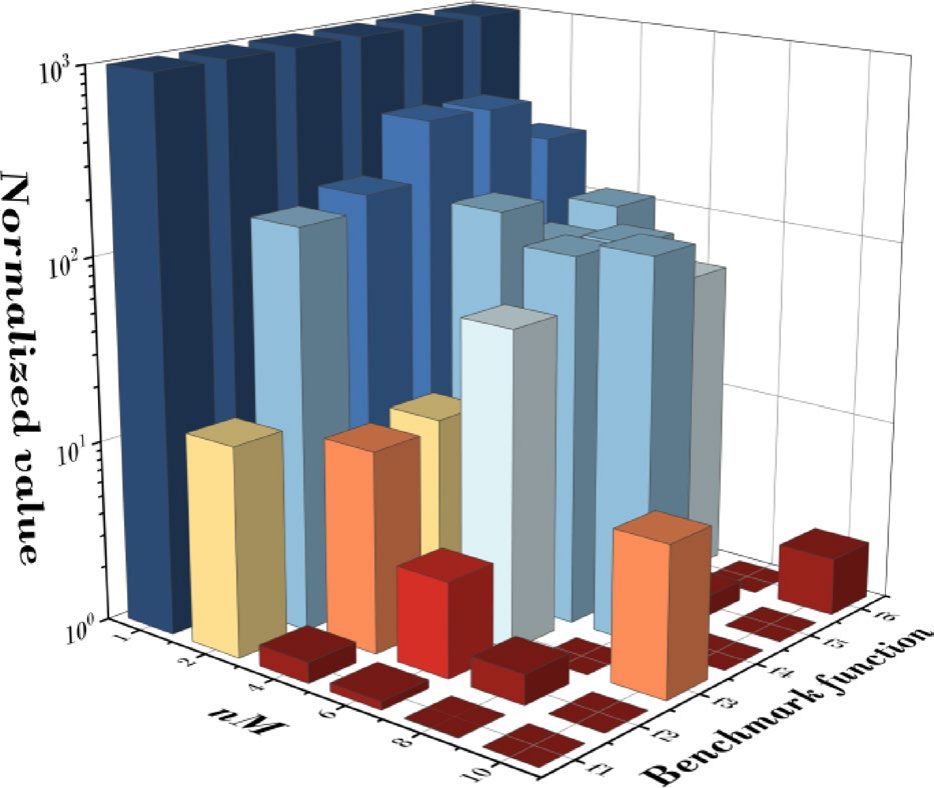
Results according to change of nM.

Figure 10; Table 8 present the statistical validation results based on the mean fitness values obtained from 20 independent runs for six benchmark functions (f1–f6) at each nM value. A smaller Friedman value indicates superior convergence performance. The analysis shows that when nM = 1, the convergence performance was the lowest for all functions, and as nM increased, the performance generally improved. In particular, the best convergence performance was observed at nM = 10, with notable improvements continuing up to nM = 8, after which the gains tended to plateau. This suggests that once the measurement frequency is sufficiently high, further increases in nM have a reduced impact on convergence. For statistical significance testing, the Friedman test was conducted, resulting in X^2^_F_ = 25.57, *p* = 1.14E-4 (*p* < 0.001), confirming that the performance differences according to nM are highly significant. Furthermore, the Page’s L test was performed to examine the monotonic trend of the rankings, yielding L = 353.0, *p* = 0.000 (*p* < 0.001), which strongly supports the finding that performance improves as nM increases. Specifically, in terms of mean fitness (M.F.), the best results for each function were 3.183E-07 for f1 at nM = 10, 1.173E-04 for f2 at nM = 10, 2.111E + 01 for f3 at nM = 8, 1.591E + 01 for f4 at nM = 10, 8.620E-04 for f5 at nM = 10, and 1.109E-01 for f6 at nM = 8.

These results indicate that increasing nM allows for more precise sampling of the qubit’s probability state, which expands the search capability and increases the likelihood of finding better solutions. However, since performance improvements diminish beyond nM = 8, selecting nM within a range that balances accuracy and computational cost may be optimal. For reference, Table A3 in the Appendix provides the detailed best fitness (B.F.), mean fitness (M.F.), and standard deviation (Std.) values for each function.

**Table 8:** Friedman value for change of nM.

nM	1	2	4	6	8	10
Friedman value	6.00	5.00	3.67	3.17	1.83	1.33
Friedman test	X^2^_F_ = 25.57, *p* = 1.14E-4					
Page’s test	L = 353.00, *p* = 0.00					

### $\:{\upepsilon\:}$

$\:{\upepsilon\:}$ is a parameter used in the H_ε_ gate, and convergence of qubits is suppressed according to $\:{\upepsilon\:}$. As ε increases, it moves away from complete convergence to 0 or 1, and as ε decreases, it approaches complete convergence. To compare convergence performance, 0.00, 0.02, 0.05, 0.07, 0.10, 0.15, 0.20, 0.30, and 0.40 were selected as values of $\:{\upepsilon\:}$, and other parameters were fixed as shown in Table 9.

**Table 9:** Parameters for $\:{\upepsilon\:}$ analysis.

System	Parameters
For CSA	Dimension of problem (d): 5 Flock size (N): 10 Awareness probability (AP): 0.1 ŸFlight length (fl.): 2
For qubits	ŸNumber of qubits (nQ): 20 ŸNumber of measurements (nM): 1 ŸRotation angle ($\:{\theta\:}_{r}$): 0.06

**Fig. 11:**
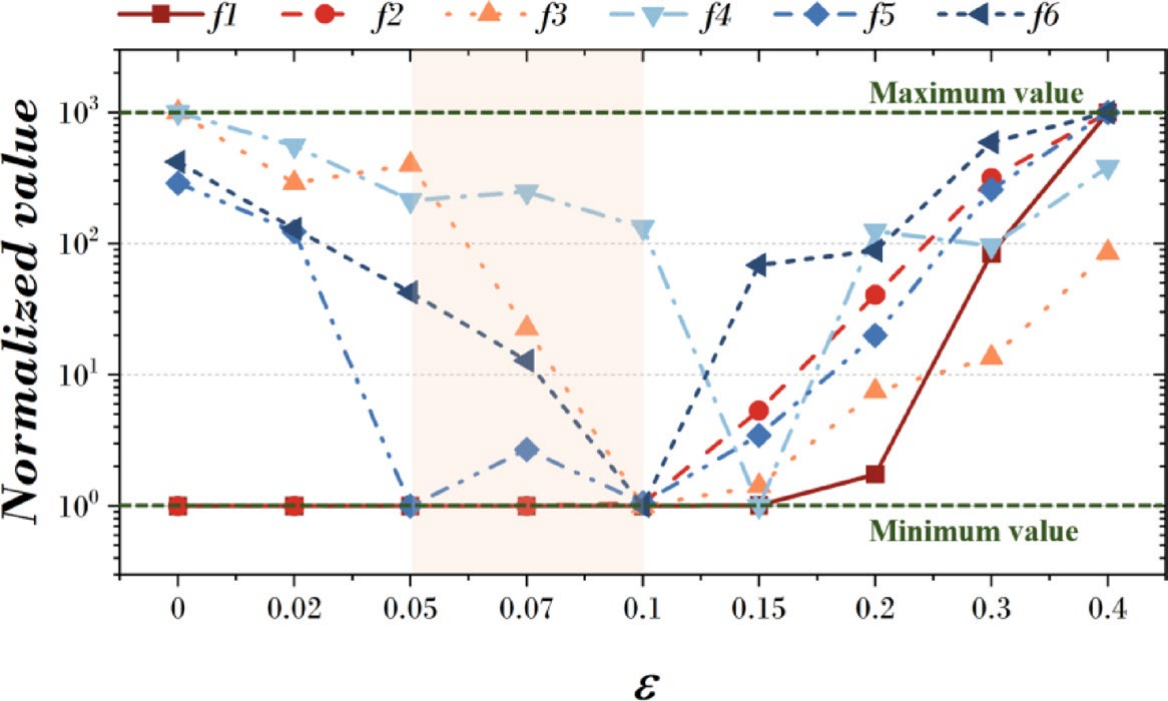
Results according to change of $\:{\upepsilon\:}$.

Figure 11; Table 10 present the statistical validation results based on the mean fitness (M.F.) values from 20 independent runs for six benchmark functions (f1–f6) at each *ε* value. The Friedman test yielded X^2^_F_ = 40.54, *p* = 3.12E-7 (*p* < 0.001), confirming that performance differences according to *ε* are highly significant. Furthermore, the Page’s L test produced L = 936.0, *p* = 0.000 (*p* < 0.001), indicating a strong monotonic trend in performance changes with respect to *ε*. In detail, f1 and f2 exhibited the best convergence at *ε* = 0.00, with M.F. values of 4.547E-08 and 4.768E-05, respectively. f3, f4, f5, and f6 showed optimal performance within a specific range of *ε*, particularly 0.05–0.10. Specifically, the best M.F. results were 1.285E-01 for f3 at *ε* = 0.10, 1.388E + 01 for f4 at *ε* = 0.15, 1.221E-04 for f5 at *ε* = 0.05, and 7.130E-02 for f6 at *ε* = 0.10. According to the Friedman ranking results in Table 10, *ε* = 0.07 and *ε* = 0.10 both achieved the lowest ranking value of 3.00, representing the best overall convergence performance.

These results suggest that the Hε gate’s effect varies depending on the problem’s landscape. For unimodal problems without local minima (e.g., f1 and f2), the gate has minimal impact, and smaller *ε* values are advantageous. For multimodal problems with many local minima (e.g., f3–f6), moderate *ε* values (0.05–0.10) help maintain exploration while guiding convergence, leading to better overall performance. In practical optimization problems where local minima are common, tuning *ε* within this range may provide a good balance between exploration and exploitation. For reference, Table A4 in the Appendix provides the detailed best fitness (B.F.), mean fitness (M.F.), and standard deviation (Std.) values for each function.

**Table 10:** Friedman value for change of $\:{\upepsilon\:}$.

$\:{\upepsilon\:}$	0.00	0.02	0.05	0.07	0.10	0.15	0.20	0.30	0.40
Friedman value	5.83	4.83	3.17	3.00	3.00	3.83	5.00	6.17	8.17
Friedman test	X^2^_F_ = 40.54, *p* = 3.12E-7								
Page’s test	L = 936.00, *p* = 0.00								

### $\:{\theta\:}_{R}$

$\:{\theta\:}_{R}$ is a parameter used in the Lookup table and directly affects the rotation of qubits for convergence. As $\:{\theta\:}_{R}$ increases, the rotation angle becomes more significant, so it converges faster, and as $\:{\theta\:}_{R}$ decreases, the rotation angle becomes smaller, so it converges slowly. To compare convergence performance, 0.00, 0.05, 0.10, 0.15, 0.20, 0.30, 0.40, and 0.50 were selected as values of $\:{\theta\:}_{R}$, and other parameters were fixed as shown in Table 11.

**Table 11:** Parameters for $\:{\theta\:}_{R}$ analysis.

System	Parameters
For CSA	ŸDimension of problem (d): 5 ŸFlock size (N): 10 ŸAwareness probability (AP): 0.1 ŸFlight length (fl.): 2
For qubits	ŸNumber of qubits (nQ): 20 ŸNumber of measurements (nM): 1 Ÿ$\:{\upepsilon\:}$ for H_ε_ gate: 0.15

**Fig. 12:**
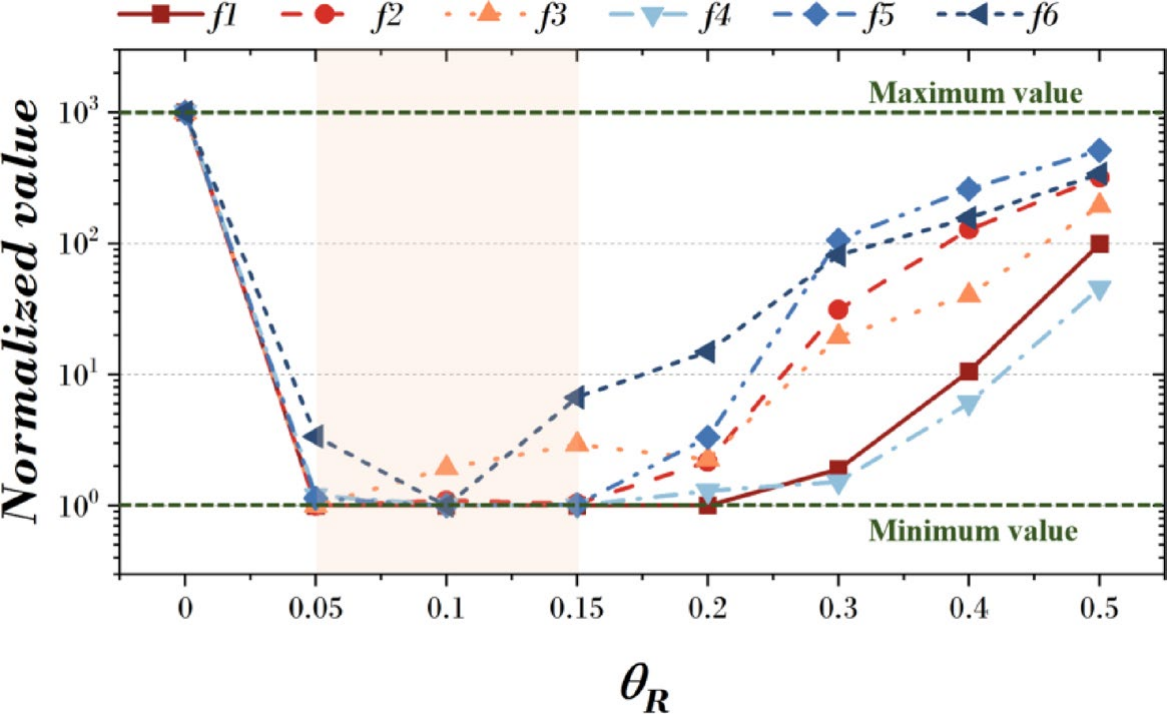
Results according to change of $\:{\theta\:}_{R}$.

Figure 12; Table 12 summarize the statistical validation results based on the mean fitness values (M.F.) obtained from 20 independent runs for six benchmark functions (f1–f6) at each $\:{\theta\:}_{R}$ value. A smaller Friedman value indicates superior convergence performance. Specifically, in terms of mean fitness (M.F.), the best results for each function were 1.326E-04 for f1 at $\:{\theta\:}_{R}$ = 0.15, 1.376E-03 for f2 at $\:{\theta\:}_{R}$ = 0.05, 1.828E-01 for f3 at $\:{\theta\:}_{R}$ = 0.05, 4.989E+01 for f4 at $\:{\theta\:}_{R}$ = 0.15, 6.157E-03 for f5 at $\:{\theta\:}_{R}$ = 0.10, and 1.017E-01 for f6 at $\:{\theta\:}_{R}$ = 0.10. Across all functions, the lowest performance was observed at $\:{\theta\:}_{R}$ = 0.00, and performance improved when $\:{\theta\:}_{R}$ was set within the range of 0.05–0.15. For statistical significance testing, the Friedman test was conducted, resulting in X^2^_F_ = 29.43, *p* = 3.8 × 10⁻⁵ (*p* < 0.001), confirming that performance differences according to $\:{\theta\:}_{R}$ are highly significant. Furthermore, Page’s L test was performed to examine the monotonic trend of rankings, yielding L = 350.0, *p* = 0.000 (*p* < 0.001), which strongly supports the finding that performance improves when $\:{\theta\:}_{R}$ is within the optimal range.

These results indicate that $\:{\theta\:}_{R}$ directly influences convergence performance through the degree of rotation applied to the qubit, and that there exists an optimal rotation angle range in which the balance between exploration and exploitation yields the best results. Selecting $\:{\theta\:}_{R}$ too small limits the search diversity, while too large may destabilize convergence. For reference, Table A5 in the Appendix provides the detailed best fitness (B.F.), mean fitness (M.F.), and standard deviation (Std.) values for each function.

**Table 12:** Friedman value for change of $\:{\theta\:}_{R}$.

$\:{\theta\:}_{R}$	0.00	0.05	0.10	0.15	0.20	0.30	0.40	0.50
Friedman value	8.00	2.00	2.00	2.17	3.83	5.00	6.00	7.00
Friedman test	X^2^_F_ = 36.27, *p* = 1.58E-6							
Page’s test	L = 320.00, *p* = 0.00							

### Result of the parameter analysis

The parameter analysis in this study was conducted using the OFAT (one factor at a time) approach, in which only the parameter under investigation was varied in each analysis, while the remaining parameters were fixed at the baseline values specified in the table. Through this procedure, recommended ranges were derived for N, nQ, nM, $\:{\upepsilon\:}$, and $\:{\theta\:}_{R}$ that consistently demonstrated favorable performance across six benchmark functions. The summary of the results for each parameter is as follows:

- N: Increasing N improves convergence stability.
- nQ: Larger values enhance convergence performance, with stable convergence observed from values of 30 and above.
- nM: Larger values increase the probability of finding high-quality solutions, thereby improving convergence performance.
- $\:{\upepsilon\:}$: Values in the range of 0.07–0.10 enhance convergence performance.
- $\:{\theta\:}_{R}$: Values in the range of 0.05–0.15 avoid over- or under-rotation, improving convergence performance.

All observed trends were statistically validated through the Friedman test and Page’s L test, with results indicating high statistical significance (*p* < 0.001). Notably, the benchmark set included both unimodal and multimodal functions, ensuring that improvements were not limited to cases favoring exploitation. In multimodal functions, which demand strong exploration to avoid premature convergence, the proposed parameter settings maintained or improved performance, indicating that the exploration–exploitation balance was preserved. These findings suggest that the derived ranges are not arbitrary but are supported by robust statistical evidence. However, a systematic analysis of the interaction effects among parameter combinations and the performance variations across different problem types was not conducted in this study. Therefore, the values presented in this section should not be regarded as universally optimal for all problems, but rather as reasonable initial settings that can be tested for a variety of problem types.

## Comparison of QbCSA performance with other algorithms

In this chapter, the performance and applicability of QbCSA to real-world engineering problems are evaluated using the CEC2019 benchmark functions and four practical engineering optimization problems. For the convergence performance comparison, the QbHSA proposed by Lee et al. was employed^42^. As described in Chap. 2, QbCSA represents design variables as qubit probabilities, updates these probabilities, and then obtains decimal values through a measurement process. Due to this structural characteristic, it is not directly comparable to most conventional metaheuristic algorithms that represent design variables directly in decimal form and adjust them continuously. However, since CSA served as the underlying inspiration for QbCSA, it was included as an additional comparison target to provide a clearer understanding of QbCSA’s characteristics. The parameter settings for each algorithm are presented in Table 13, and all analyses were performed under identical conditions with 20 independent runs.

**Table 13:** Parameters for each algorithms.

Algorithm	Parameters
QbCSA	d: Defend on problem; N: 10; AP: 0.1; fl.: 2; nQ: 30; nM: 1; $\:{\upepsilon\:}$: 0.10; $\:{\theta\:}_{R}$: 0.06; $\:\gamma\:$: 1.00
QbHSA	d: Defend on problem; N: 10; Q-HMCR: 0.9; Q-PAR: 0.1; tolBWpa: 0.95; BWQ: 0.3; BWQ_max: 1.0; BWQ_min: 0.1; nQ: 30; nM: 1; $\:{\upepsilon\:}$: 0.10; $\:{\theta\:}_{R}$: 0.06; $\:\gamma\:$: 1.00
CSA	d: Defend on problem; N: 10; AP: 0.1; fl.: 2.0

### Results for CEC2019

For the comparison of the convergence performance of each algorithm, the CEC 2019 benchmark functions are presented in Table 14^43^. The CEC 2019 function set consists of ten multimodal functions and is designed to evaluate the behavioral characteristics of optimization algorithms in detail. Each algorithm was executed independently 20 times to obtain the minimum fitness values, from which the global minimum average and global minimum standard deviation were calculated. The ten benchmark functions can be categorized into two groups according to their structural characteristics. F1–F3 (Non-rotated functions) do not involve rotation or shifting, making them relatively simple in structure but exhibiting strong inter-variable dependencies. F4–F10 (Rotated and shifted functions) are highly complex multimodal problems with numerous local optima and intricate variable interactions, thereby posing significant challenges to optimization.

**Table 14:** CEC 2019 benchmark functions.

No,	Functions	d	Boundary	Best
F1	Storn’s Chebyshev Polynomial Fitting Problem	9	[-8192 8192]	1
F2	Inverse Hilbert Matrix Problem	16	[-16,384 16,384]	1
F3	Lennard-Jones Minimum Energy Cluster	18	[-4 4]	1
F4	Rastrigin’s Function	10	[-100 100]	1
F5	Griewangk’s Function	10	[-100 100]	1
F6	Weierstrass Function	10	[-100 100]	1
F7	Modified Schwefel’s Function	10	[-100 100]	1
F8	Expanded Schaffer’s F6 Function	10	[-100 100]	1
F9	Happy Cat Function	10	[-100 100]	1
F10	Ackley Function	10	[-100 100]	1

Tables 15 and 16 summarize the optimization performance and the Wilcoxon rank-sum test results for the proposed QbCSA, QbHSA, and conventional CSA on the CEC2019 benchmark functions. For F1–F3, both QbCSA and QbHSA achieved optimization performance comparable to CSA. In particular, for F2 and F3, QbCSA recorded Best and Mean values close to QbHSA, while its standard deviation (Std) was slightly lower, indicating relatively stable convergence. However, according to the Wilcoxon rank-sum test, the performance difference between QbCSA and QbHSA was not statistically significant (*p* ≥ 0.05), suggesting that both algorithms exhibit similar search behaviors in simple search spaces. For F4–F10, different performance trends were observed depending on problem complexity. For F4, F5, F6, and F8, both QbCSA and QbHSA achieved Best values similar to CSA, and no statistically significant differences were found between the two quantum-based methods. However, F7, characterized by a large number of local optima and high sensitivity to noise, showed that QbHSA exhibited slightly more stable convergence than QbCSA in terms of standard deviation, although this difference was not statistically significant. On the other hand, comparisons between QbCSA and CSA revealed distinct differences depending on problem difficulty.

According to the Wilcoxon rank-sum test (Table 16), QbCSA significantly outperformed CSA for F6 and F7 (*p* < 0.05), indicating that qubit-based variable encoding can enhance the search efficiency of CSA in complex multimodal landscapes. In contrast, CSA showed better results than QbCSA in F1, F2, F3, F4, and F9, while no significant differences were observed for F5, F8, and F10. Statistical analysis from Table 16 shows that QbCSA and QbHSA exhibited no significant performance differences across most benchmark functions, and QbCSA maintained overall performance levels comparable to CSA. Nevertheless, the statistically significant superiority of QbCSA on complex problems (F6, F7) underscores the academic relevance of the proposed method. Therefore, QbCSA is not positioned as a fundamentally new optimization algorithm but rather as an analytical framework that empirically demonstrates how qubit-based design variables can alter the search dynamics of CSA. This provides practical insights into integrating quantum-inspired variable representations into existing metaheuristic approaches.

These results indicate that while QbCSA does not consistently outperform CSA in simpler optimization problems, its statistically significant superiority in complex multimodal functions (F6, F7) highlights the potential advantage of qubit-based encoding in enhancing exploration efficiency. Therefore, the primary contribution of this study lies in demonstrating how quantum-inspired variable representations can improve the convergence behavior of metaheuristic algorithms in challenging optimization landscapes.

**Table 15:** Minimum fitness on CEC2019 benchmark functions (bold values indicate the best results for each metric).

No.	Metrix	QbCSA	QbHSA	CSA
F1	Best	2.323E + 08	3.100E + 08	**4.386E + 06**
	Mean	5.087E + 09	5.725E + 09	**9.095E + 07**
	Std	4.647E + 09	6.474E + 09	**7.062E + 07**
F2	Best	**1.734E + 01**	1.768E + 01	**1.734E + 01**
	Mean	1.851E + 01	1.879E + 01	**1.734E + 01**
	Std	7.418E-01	6.505E-01	**4.228E-11**
F3	Best	**1.270E + 01**	**1.270E + 01**	**1.270E + 01**
	Mean	**1.270E + 01**	**1.270E + 01**	**1.270E + 01**
	Std	2.707E-05	3.051E-05	**3.645E-15**
F4	Best	3.756E + 01	3.145E + 01	**3.084E + 01**
	Mean	1.456E + 02	1.642E + 02	**8.765E + 01**
	Std	6.328E + 01	8.542E + 01	**2.959E + 01**
F5	Best	1.119E + 00	1.182E + 00	**1.081E + 00**
	Mean	**1.315E + 00**	1.366E + 00	1.342E + 00
	Std	1.458E-01	**1.276E-01**	2.460E-01
F6	Best	**2.387E + 00**	3.131E + 00	4.518E + 00
	Mean	**4.179E + 00**	4.604E + 00	7.028E + 00
	Std	**7.533E-01**	7.630E-01	1.666E + 00
F7	Best	1.999E-01	3.564E-01	**1.273E-04**
	Mean	**5.498E + 00**	2.406E + 01	1.206E + 01
	Std	**3.994E + 00**	6.122E + 01	5.295E + 01
F8	Best	3.483E + 00	**3.093E + 00**	3.784E + 00
	Mean	5.008E + 00	5.189E + 00	**4.899E + 00**
	Std	**6.737E-01**	7.959E-01	6.823E-01
F9	Best	2.490E + 00	2.764E + 00	**2.339E + 00**
	Mean	4.293E + 00	3.995E + 00	**2.342E + 00**
	Std	9.874E-01	8.753E-01	**2.311E-03**
F10	Best	**2.000E + 01**	**2.000E + 01**	**2.000E + 01**
	Mean	**2.002E + 01**	**2.002E + 01**	2.009E + 01
	Std	1.868E-02	**1.370E-02**	9.819E-02

Significant values are in bold.

**Table 16:** Wilxocon rank-sum test of QbCSA and its competitors on CEC 2019 benchmark functions.

No.	QbHSA	CSA
F1	–	–
F2	=	–
F3	=	–
F4	=	–
F5	=	=
F6	=	+
F7	=	+
F8	=	=
F9	=	–
F10	=	=
Statistics number (+/–/=)	0/1/9	2/5/3

**Fig. 13:**
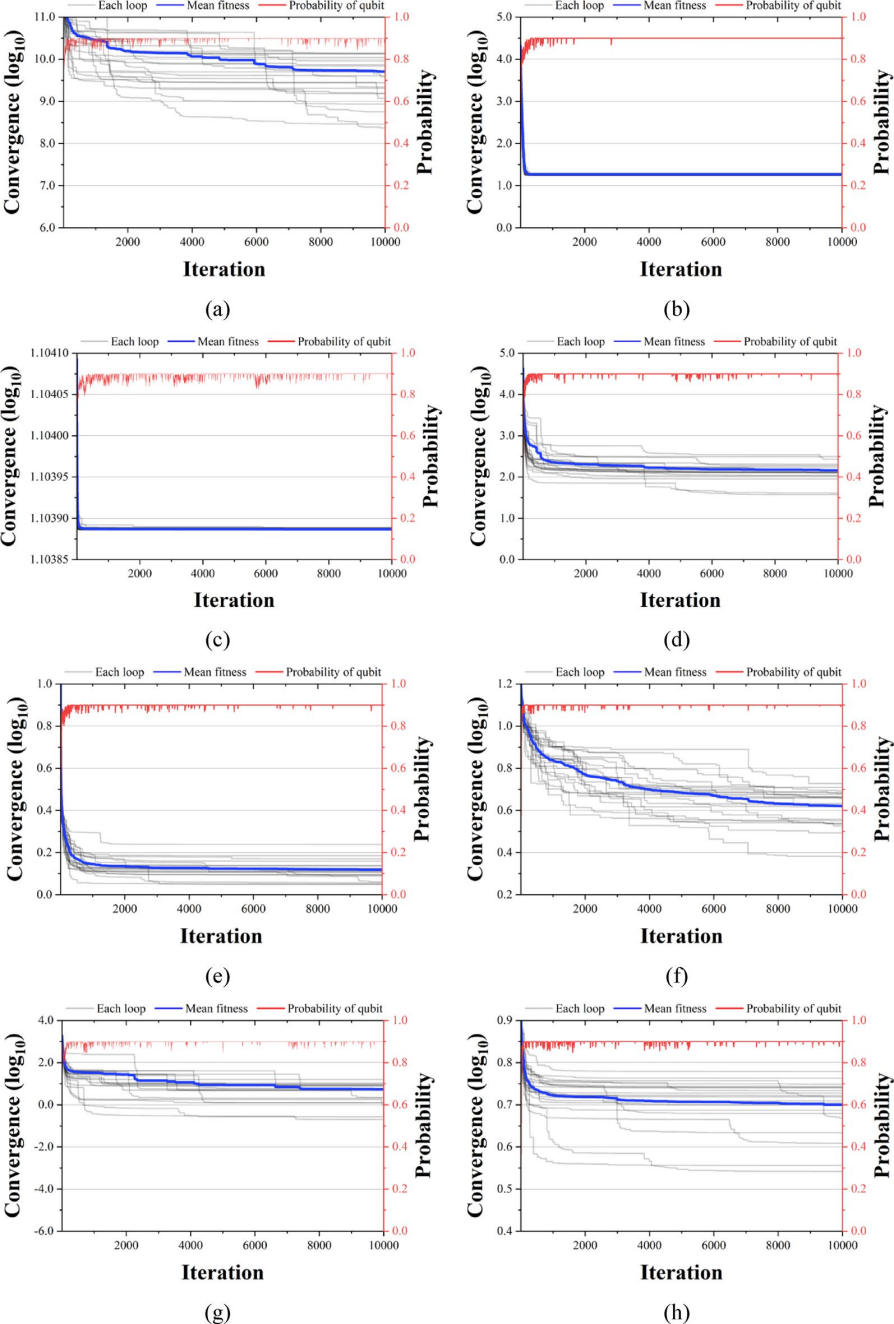

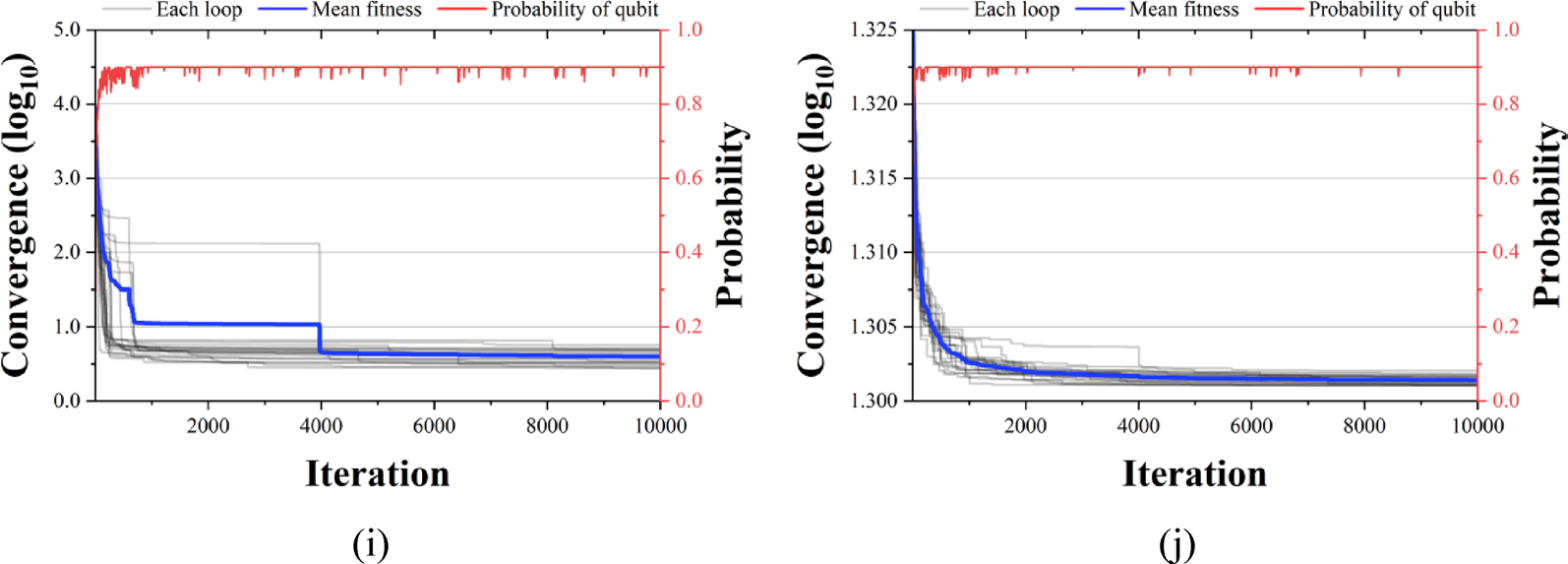
Convergence graph of QbCSA. (**a**) F1; (**b**) F2; (**c**) F3; (**d**) F4; (**e**) F5; (**f**) F6; (**g**) F7; (**h**) F8; (**i**) F9; (**j**) F10.

**Fig. 14:**
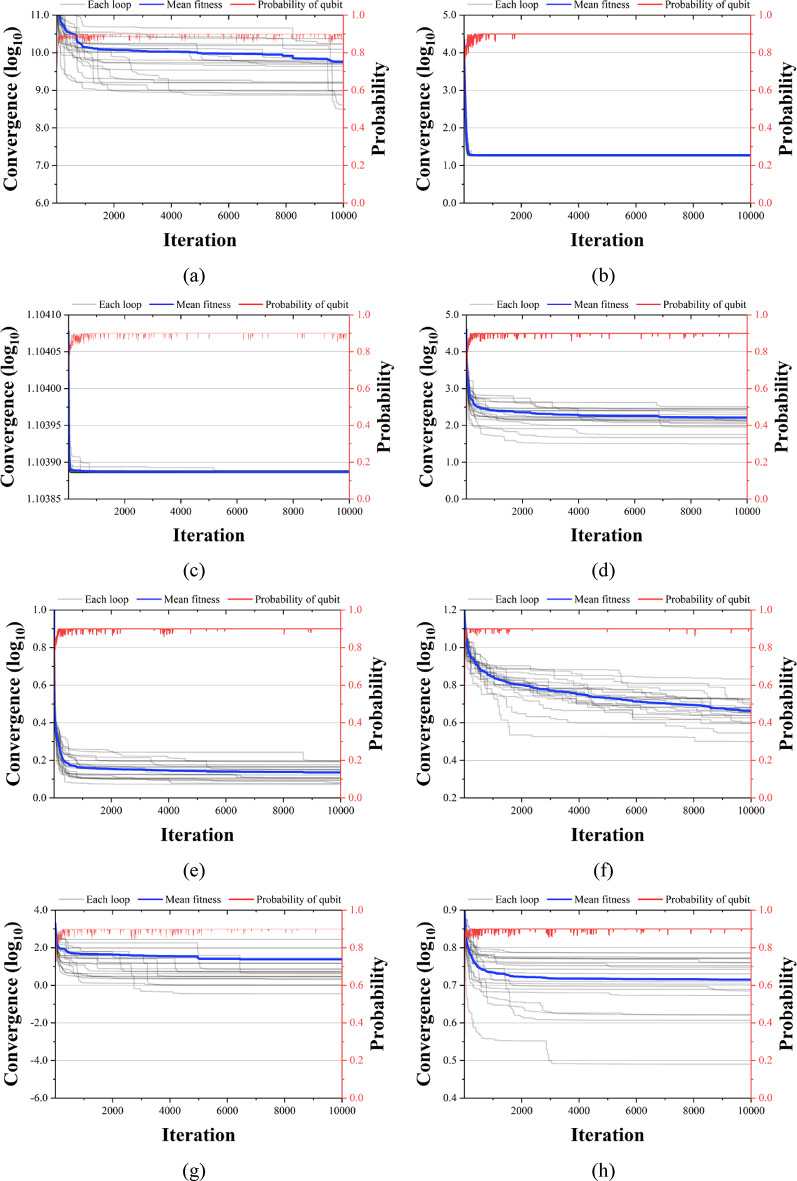

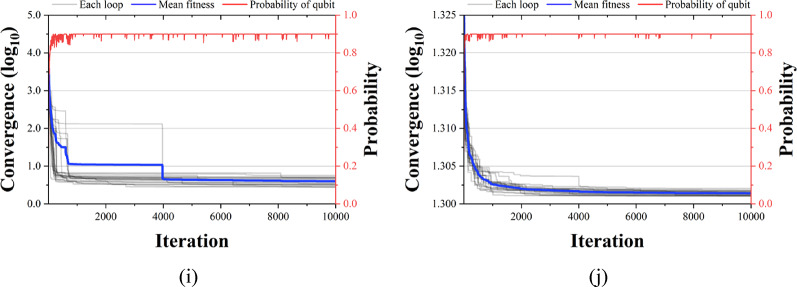
Convergence graph of QbHSA. (**a**) F1; (**b**) F2; (**c**) F3; (**d**) F4; (**e**) F5; (**f**) F6; (**g**) F7; (**h**) F8; (**i**) F9; (**j**) F10.

**Fig. 15:**
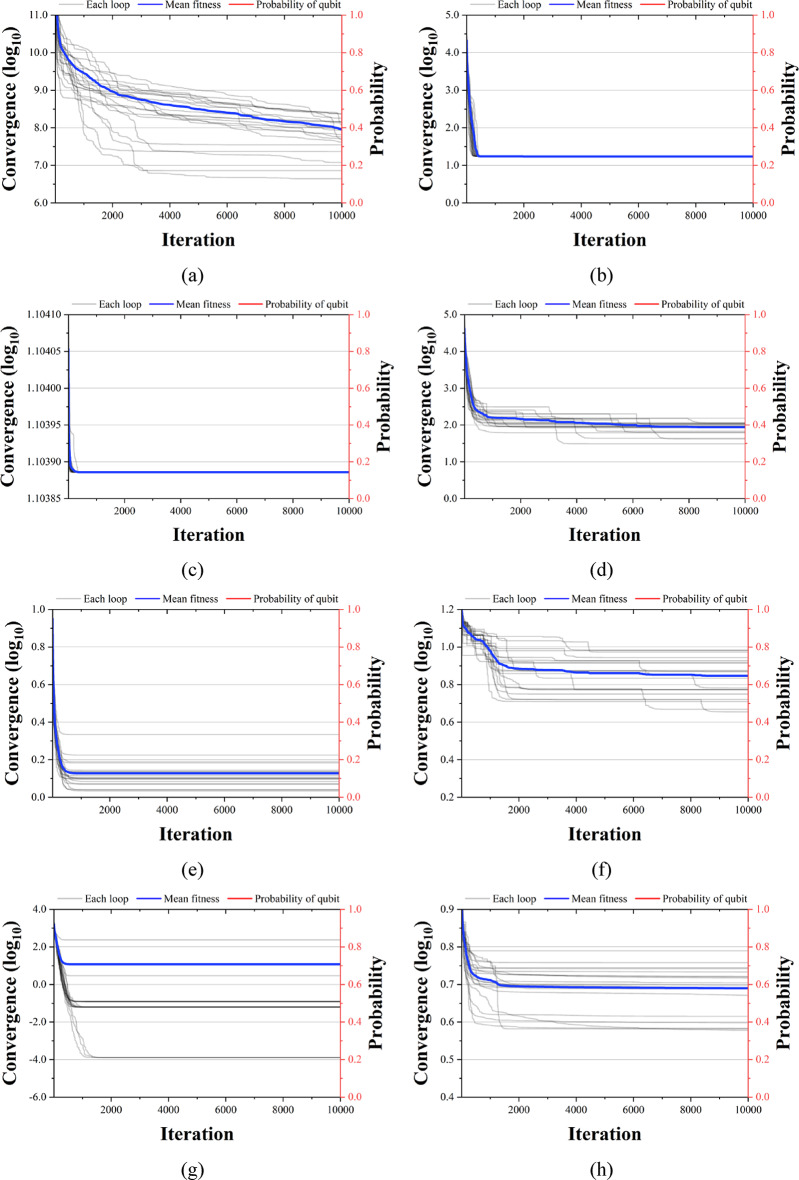

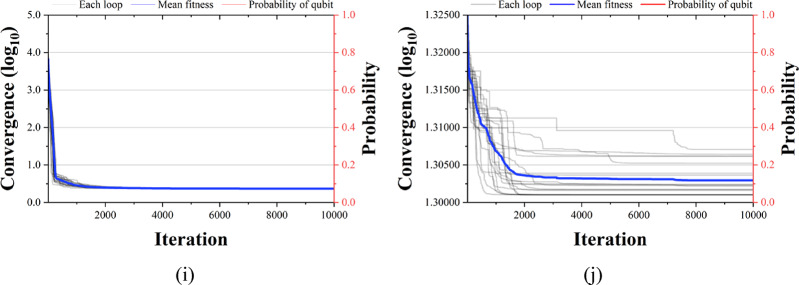
Convergence graph of CSA. (**a**) F1; (**b**) F2; (**c**) F3; (**d**) F4; (**e**) F5; (**f**) F6; (**g**) F7; (**h**) F8; (**i**) F9; (**j**) F10.

Figures 13, 14 and 15 compare the convergence performance of QbCSA, QbHSA, and CSA on the CEC2019 benchmark functions (F1–F10). In each figure, the blue solid line represents the average fitness, the gray lines indicate the fitness evolution of individual runs, and the red line shows the qubit probability evolution. For the non-rotated functions (F1–F3), particularly F2 and F3, all three algorithms demonstrate a rapid convergence toward the global optimum. In contrast, for the rotated and shifted functions (F4–F10), the increased problem complexity leads to larger performance differences among the algorithms, with QbCSA exhibiting superior global search capability over CSA for several functions. Additionally, it is observed that the qubit values used as design variables in QbCSA and QbHSA converge toward approximately 0.9 ($\:{\upepsilon\:}$ = 0.1).

### Engineering problem

#### PVD (pressure vessel design) problem

**Fig. 16:**
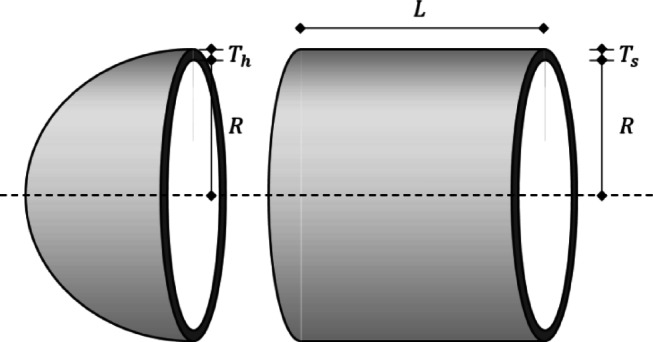
PVD problem.

The PVD problem uses shell thickness ($\:{T}_{s}$; $\:{x}_{1}$), head thickness ($\:{T}_{h}$; $\:{x}_{2}$), inner radius ($\:R$; $\:{x}_{3}$), and container length ($\:L$; $\:{x}_{4}$) as design variables and aims to minimize material, forming, and welding costs defined by Fig. 16 and Eq. (14). $\:{T}_{s}$ and $\:{T}_{h}$ can range from 0 to 100, and $\:R$ and $\:L$ can range from 10 to 200.

14 $$Minimize\,\,\,f\left( x \right) = 0.6224x_{1} x_{3} x_{4} + 1.7781x_{2} x_{3}^{2} + 3.1661x_{1}^{2} x_{4} + 19.84x_{1}^{2} x_{3}$$

Subject to: $$\:{g}_{1}\left(x\right)=-{x}_{1}+0.0193{x}_{3}\le\:0$$

$$\:{g}_{2}\left(x\right)=-{x}_{2}+0.00954{x}_{3}\le\:0$$

$$\:{g}_{3}\left(x\right)=-\pi\:{x}_{3}^{2}{x}_{4}-\frac{4}{3}\pi\:{x}_{3}^{3}+\text{1,296,000}\le\:0$$

$$\:{g}_{4}\left(x\right)=-{x}_{4}+240\le\:0$$

Table 17 shows the PVD problem and compares it with the results of previous studies. The previous studies, QbCSA and QbHSA, drew results that satisfied all the constraints. In the previous study, 6410.381, 7198.043, and 8129.104 were derived, and QbHSA was 6422.613. QbCSA derives design variables of 0.8809, 0.4050, 42.2664, and 175.0125, showing the best fitness at 6422.613. In addition, compared to QbHSA using the same quantum system, an improvement of about 0.655% was derived.

**Table 17:** Results of PVD problem (bold values indicate the best results for each metric).

Design variables	Deb^44^	Kannan & Kramer^45^	Sandgren^46^	This paper	
				QbCSA	QbHSA
$\:{x}_{1}$	0.9375	1.125	1.125	0.9907	0.8809
$\:{x}_{2}$	0.5000	0.625	0.625	0.4904	0.4050
$\:{x}_{3}$	48.3290	58.291	47.700	51.2463	42.2664
$\:{x}_{4}$	112.6790	43.690	117.701	89.1180	175.0125
$\:{g}_{1}\left(x\right)$	-0.0048	0.0000	-0.2044	-0.0016	-0.0625
$\:{g}_{2}\left(x\right)$	-0.0389	-0.0689	-0.1699	-0.0015	-0.0018
$\:{g}_{3}\left(x\right)$	-3,652.8768	-21.2201	54.2260	-2,995.4713	-2,503.4852
$\:{g}_{4}\left(x\right)$	-127.3210	-196.3100	-122.2990	-150.8820	-64.9875
Result	6,410.381	7,198.043	8,129.104	**6**,**380.825**	6,422.613

Significant values are in bold.

#### WBD (welded beam design) problem

**Fig. 17:**
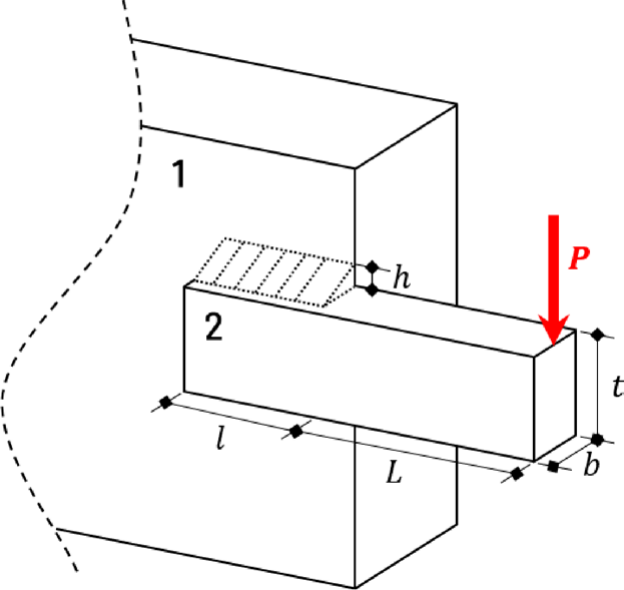
WBD problem.

The WBD problem uses welding height ($\:h$; $\:{x}_{1}$), welding length ($\:l$; $\:{x}_{2}$), thickness of beam ($\:t$; $\:{x}_{3}$), and width of beam ($\:b$; $\:{x}_{4}$) as design variables and aims to minimize welding and material costs defined by Fig. 17 and Eq. (15). $\:h$ and $\:b$ can range from 0.1 to 2, and $\:l$ and $\:t$ can range from 0.1 to 10. For the constraints calculation, $\:P$ is 6000 lb, $\:L$ is 14.0 inches, $\:E$ (modulus of elasticity) is 30 × 10^6^ psi, $\:G$ (modulus of shear elasticity) is 12 × 10^6^ psi, $\:{\tau\:}_{max}$ (maximum shear stress) is 13,600 psi, $\:{\sigma\:}_{max}$ (maximum stress) is 30,000 psi, and $\:{\delta\:}_{max}$ (maximum displacement) is 0.25 inches.

15 $$Minimize\,\,\,f\left( x \right) = 1.10471x_{1}^{2} x_{2} + 0.04811x_{3} x_{4} \left( {14.0 + x_{2} } \right)$$

Subject to: $$\:{g}_{1}\left(x\right)=\tau\:\left(x\right)-{\tau\:}_{max}\le\:0$$

$$\:{g}_{2}\left(x\right)=\delta\:\left(x\right)-{\delta\:}_{max}\le\:0$$

$$\:{g}_{3}\left(x\right)={x}_{1}-{x}_{4}\le\:0$$

$$\:{g}_{4}\left(x\right)=0.10471{x}_{1}^{2}+0.04811{x}_{3}{x}_{4}\left(14.0+{x}_{2}\right)-5.0\le\:0$$

$$\:{g}_{5}\left(x\right)=0.125-{x}_{1}\le\:0$$

$$\:{g}_{6}\left(x\right)=\delta\:\left(x\right)-{\delta\:}_{max}\le\:0$$

$$\:{g}_{7}\left(x\right)=P-{P}_{x}\left(x\right)\le\:0$$

Table 18 shows the result of the WBD problem and compares it with the results of previous studies. Both previous studies and QbCSA and QbHSA drew results that satisfy the constraints. In previous studies, 2.431, 2.3815, and 2.3859 were derived, and QbHSA 2.0600 was derived. QbCSA derived design variables of 0.2390, 3.1938, 8.1438, and 0.2547, showing the best convergence performance at 1.9172. In addition, a result of about 7.448% improvement compared to QbHSA was derived.

**Table 18:** Results of welded beam design problem (bold values indicate the best results for each metric).

Design variables	Deb^47^	Siddall^48^	Ragsdell & Phillips^49^	This paper	
				QbCSA	QbHSA
$\:{x}_{1}$	0.2489	0.2444	0.2455	0.2390	0.1689
$\:{x}_{2}$	6.1730	6.2189	6.1960	3.1938	5.0500
$\:{x}_{3}$	8.1789	8.2915	8.2730	8.1438	8.1438
$\:{x}_{4}$	-0.2533	0.2444	0.2455	0.2547	0.2547
$\:{g}_{1}\left(x\right)$	-5,758.6038	-5,743.5020	-5,743.8265	-31.8343	-31.8523
$\:{g}_{2}\left(x\right)$	-255.5769	-4.0152	-4.7151	-163.5332	-163.5332
$\:{g}_{3}\left(x\right)$	-0.0044	0.0000	0.0000	-0.0157	-0.0858
$\:{g}_{4}\left(x\right)$	-2.9829	-3.0226	-3.0203	-3.2782	-3.0960
$\:{g}_{5}\left(x\right)$	-0.1239	-0.1194	-0.1205	-0.1140	-0.0439
$\:{g}_{6}\left(x\right)$	-0.2342	-0.2342	-0.2342	-0.2340	-0.2340
$\:{g}_{7}\left(x\right)$	-4,465.2709	-3,490.4694	-3,604.2750	-4,607.7486	-4,607.7486
Result	2.4331	2.3815	2.3859	**1.9172**	2.0600

Significant values are in bold.

#### 3-bar truss optimization problem

**Fig. 18:**
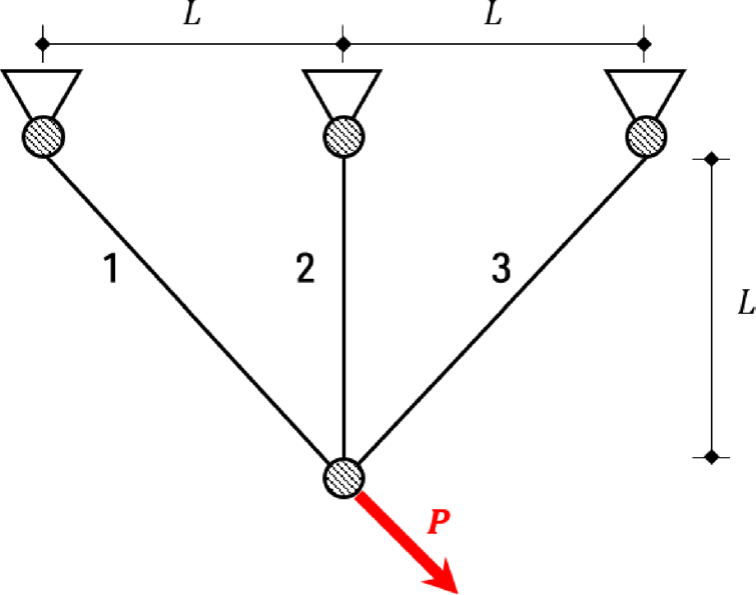
3-bar truss optimization problem.

The 3-bar truss optimization problem has the cross-sectional area of element 1 ($\:{A}_{1}$; $\:{x}_{1}$) and element 2 ($\:{A}_{2}$; $\:{x}_{2}$) as design variables. Elements 1 and 3 are assumed to have the same cross-sectional area. This problem aims to minimize the weight using the cross-sectional area of three members defined by Fig. 18 and Eq. (16). $\:{A}_{1}$ and $\:{A}_{2}$ may have a range from 0.0 to 1.0. For the constraints calculation, $\:L$ is 100 cm, $\:P$ is 2 kN/cm^2^, and $\:{\sigma\:}_{max}$ is 2 kN/cm^2^.

16 $$Minimize\,\,\,f\left( x \right) = \left( {2\sqrt {2x_{1} } + x_{2} } \right)l$$

Subject to: $$\:{g}_{1}\left(x\right)=\frac{\sqrt{2}{x}_{1}+{x}_{2}}{\sqrt{2}{x}_{1}^{2}+2{x}_{1}{x}_{2}}P-\sigma\:\le\:0$$

$$\:{g}_{2}\left(x\right)=\frac{{x}_{2}}{\sqrt{2}{x}_{1}^{2}+2{x}_{1}{x}_{2}}P-\sigma\:\le\:0$$

$$\:{g}_{3}\left(x\right)=\frac{1}{\sqrt{2}{x}_{2}+{x}_{1}}P-\sigma\:\le\:0$$

Table 19 shows the result of the 3-bar truss optimization problem compared with the results of previous studies. QbCSA and QbHSA satisfied all the constraints, and previous studies only expressed the results because the constraints were not expressed. In previous studies, 263.8958 and 263.8959 were derived, and QbHSA 263.9181 was derived. For QbCSA, design variables of 0.7859 and 0.4160 were used, and the result of 263.9017 was derived. In this problem, previous studies produced better results than QbCSA, but better results by 0.0164 compared to QbHSA.

**Table 19:** Results of 3-bar truss optimization problem (bold values indicate the best results for each metric).

Design variables	Askarzadeh^25^			This paper	
	SoC	MB	DSS-MDE	QbCSA	QbHSA
$\:{x}_{1}$	-	-	-	0.7859	0.7832
$\:{x}_{2}$	-	-	-	0.4160	0.4239
$\:{g}_{1}\left(x\right)$	-	-	-	-2.6653e-06	-3.6960e-07
$\:{g}_{2}\left(x\right)$	-	-	-	-1.4553	-1.4464
$\:{g}_{3}\left(x\right)$	-	-	-	-0.5446	-0.5535
Result	**263.8958**	263.8959	**263.8958**	263.9017	263.9181

Significant values are in bold.

#### SCBD (stepped cantilever beam design) problem

**Fig. 19:**
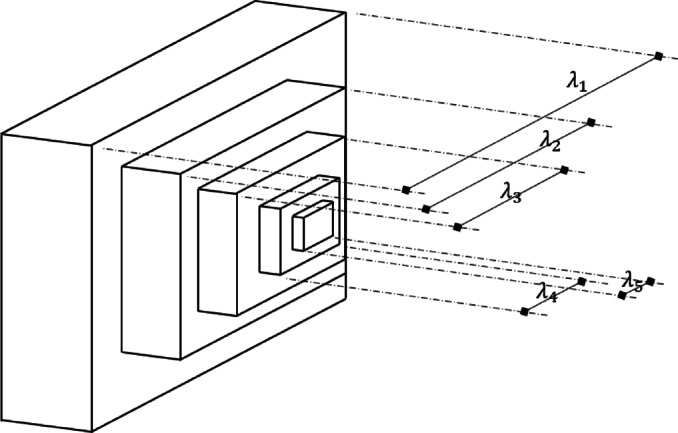
SCBD problem.

The SCBD problem uses the widths of five beams ($\:{\lambda\:}_{1-5}$; $\:{x}_{1-5}$) as design variables and aims to minimize the weight of the five beams defined by Fig. 19 and Eq. (17). $\:{\lambda\:}_{1-5}$ can range from 0.01 to 100.0.

17 $$Minimize\,\,\,f\left( x \right) = 0.0624\mathop \sum \limits_{{i = 1}}^{5} x_{i}$$

Subject to: $$\:{g}_{1}\left(x\right)=\frac{61}{{x}_{1}^{3}}+\frac{37}{{x}_{2}^{3}}+\frac{19}{{x}_{3}^{3}}+\frac{7}{{x}_{4}^{3}}+\frac{1}{{x}_{5}^{3}}-1\le\:0$$

Table 20 shows the SCBD problem and compares it with the results of previous studies. Among the preceding studies, using HHO, QbCSA, and QbHSA resulted in results satisfying the constraints. The results of AOACS and PSO in the previous study do not satisfy the constraints due to the error of decimal points. In the previous study, 1.34 and 1.38 were derived, and 1.3481 was derived for QbHSA. For QbCSA, the design variables were derived as 6.0397, 5.4782, 4.2685, 3.4734, and 2.2591, and the result of 1.3428 was derived. Although accurate comparison is difficult due to the decimal point error in the results of previous studies, it is judged that similar results are derived. In addition, the result was improved by about 0.395% compared to QbHSA.

**Table 20:** Results of SCBD problem (bold values indicate the best results for each metric).

Design variables	Hijjawi et al.^50^			This paper	
	AOACS	HHO	PSO	QbCSA	QbHSA
$\:{x}_{1}$	6.01	5.13	6.05	6.0397	5.7448
$\:{x}_{2}$	5.31	5.62	5.26	5.4782	5.2097
$\:{x}_{3}$	4.49	5.10	4.51	4.2685	4.8924
$\:{x}_{4}$	3.50	3.93	3.46	3.4734	3.4033
$\:{x}_{5}$	2.15	2.32	2.19	2.2591	2.3535
$\:{g}_{1}\left(x\right)$	0.0019	-0.0011	0.0010	-7.7574e-08	-5.1316e-05
Result	**1.34**	1.38	**1.34**	1.3428	1.3481

Significant values are in bold.

## Conclusion

In this study, we proposed QbCSA, a novel quantum-inspired metaheuristic algorithm that integrates the crow search algorithm (CSA) with qubit-based probabilistic encoding. Unlike conventional metaheuristic algorithms, which represent design variables directly in decimal form, QbCSA encodes solutions as qubit probability amplitudes and derives real values through measurement and decoding. This unique representation enhances global search efficiency and improves stability, particularly in solving complex multimodal problems. Through six benchmark functions and four engineering case studies, QbCSA demonstrated performance comparable to CSA on unimodal problems and superior convergence stability with lower variance on highly multimodal problems. Furthermore, we statistically derived recommended ranges for key quantum-related parameters—such as qubit count, measurement frequency, and rotation angle—that consistently yield favorable optimization performance. However, QbCSA introduces additional parameters compared to CSA and exhibits sensitivity to parameter configurations. To address this, adaptive parameter tuning strategies are expected to improve generalization across problem types. Future studies should continue refining the algorithm and extending its application to diverse engineering optimization domains, thereby enhancing its robustness and demonstrating the potential of quantum-inspired computation in real-world problems.

## Future works

Although QbCSA demonstrated statistically significant improvements in convergence performance and practical applicability, several important research directions remain for future studies. First, the parameter analysis in this study was performed using the one-factor-at-a-time (OFAT) approach, which does not account for the potential interactions among parameters. To better understand the influence of multiple parameters simultaneously, future work should employ multifactorial experimental designs or global sensitivity analyses to identify optimal parameter combinations and improve the algorithm’s generalizability. In addition, while QbCSA has shown promising results for six benchmark functions and four engineering examples, its performance has yet to be verified on large-scale, real-world NP-hard optimization problems. Applying QbCSA to such complex engineering tasks would provide a more comprehensive assessment of its robustness and scalability. Furthermore, the current implementation was carried out in a quantum-inspired simulation environment. Testing QbCSA on actual quantum computing hardware would be an essential next step to validate its feasibility under realistic computational constraints and evaluate its potential advantages when combined with quantum acceleration. Finally, future research should focus on enhancing the algorithmic framework itself. Incorporating more advanced qubit rotation schemes, adaptive measurement strategies, and dynamic probability amplitude control could further exploit the probabilistic nature of quantum computing, leading to additional performance gains. Moreover, integrating QbCSA into hybrid optimization frameworks or combining it with other metaheuristic algorithms could broaden its applicability and improve global search capabilities across diverse engineering and scientific domains. These directions will not only strengthen the practicality of QbCSA but also highlight the potential of quantum-inspired computation for solving increasingly complex optimization problems.

## Data Availability

All data generated or analyzed during this study are included in this published article.

## Convergence performance according to parameter changes

**Table A1:** Results according to change of N (bold values indicate the best results for mean fitness).

Function	Contents	N								
		1	5	10	15	20	25	30	40	50
F1	B.F.	1.765E-04	1.182E-07	4.547E-08	4.547E-08	4.547E-08	4.547E-08	4.547E-08	4.547E-08	4.547E-08
	M.F.	1.497E-02	1.066E-05	1.082E-06	6.730E-08	5.275E-08	**4.547E-08**	**4.547E-08**	**4.547E-08**	**4.547E-08**
	Std.	3.295E-02	1.593E-05	2.192E-06	5.196E-08	2.183E-08	1.323E-23	1.323E-23	1.323E-23	1.323E-23
F2	B.F.	4.864E-04	6.676E-05	4.768E-05	4.768E-05	4.768E-05	4.768E-05	4.768E-05	4.768E-05	4.768E-05
	M.F.	1.204E-02	2.365E-04	6.580E-05	5.913E-05	4.864E-05	**4.768E-05**	**4.768E-05**	**4.768E-05**	**4.768E-05**
	Std.	8.231E-03	2.132E-04	1.756E-05	2.208E-05	4.157E-06	0.000E + 00	0.000E + 00	0.000E + 00	0.000E + 00
F3	B.F.	1.864E + 00	9.923E-06	8.403E-05	3.911E-07	6.366E-08	6.366E-08	7.913E-07	2.728E-08	6.366E-08
	M.F.	3.541E + 02	6.267E + 00	8.515E + 00	1.246E + 00	1.009E-01	6.325E-02	6.656E-02	6.235E-02	**7.295E-05**
	Std.	5.111E + 02	1.750E + 01	2.018E + 01	4.328E + 00	2.837E-01	2.656E-01	2.675E-01	2.658E-01	2.597E-04
F4	B.F.	1.804E + 00	3.762E + 00	3.108E + 00	3.222E + 00	1.254E + 00	2.228E + 00	2.777E + 00	1.831E + 00	1.097E + 00
	M.F.	2.081E + 02	1.571E + 02	1.257E + 02	8.821E + 01	7.009E + 01	5.674E + 01	4.417E + 01	5.115E + 01	**3.639E + 01**
	Std.	4.167E + 02	2.251E + 02	3.142E + 02	1.660E + 02	1.101E + 02	1.245E + 02	9.414E + 01	1.009E + 02	8.364E + 01
F5	B.F.	4.243E-02	1.221E-04	1.221E-04	1.221E-04	1.221E-04	1.221E-04	1.221E-04	1.221E-04	1.221E-04
	M.F.	1.046E + 00	2.320E-03	3.636E-04	1.536E-04	**1.221E-04**	**1.221E-04**	**1.221E-04**	**1.221E-04**	**1.221E-04**
	Std.	1.117E + 00	3.439E-03	3.042E-04	8.983E-05	0.000E + 00	0.000E + 00	0.000E + 00	0.000E + 00	0.000E + 00
F6	B.F.	5.445E-02	1.240E-02	7.474E-03	1.663E-02	3.742E-07	7.412E-03	1.479E-02	3.742E-07	1.490E-02
	M.F.	2.486E-01	1.286E-01	8.557E-02	5.641E-02	6.684E-02	5.175E-02	4.478E-02	4.587E-02	**3.803E-02**
	Std.	1.535E-01	6.790E-02	5.246E-02	2.504E-02	4.503E-02	3.704E-02	2.580E-02	2.625E-02	1.469E-02

Significant values are in bold.

**Table A2:** Results according to change of nQ ﻿(bold values indicate the best results for mean fitness).

Function	Contents	nQ								
		1	3	5	10	15	20	30	40	50
F1	B.F.	5.000E + 04	1.020E + 03	5.203E + 01	4.778E-02	4.657E-05	4.547E-08	7.835E-10	5.988E-10	2.014E-09
	M.F.	5.000E + 04	1.020E + 03	5.203E + 01	4.778E-02	4.657E-05	2.092E-07	**1.259E-07**	3.250E-07	1.855E-07
	Std.	0.000E + 00	3.938E-13	1.204E-13	2.896E-15	1.364E-20	2.990E-07	1.448E-07	5.546E-07	1.823E-07
F2	B.F.	1.001E + 05	1.309E + 01	1.616E + 00	4.888E-02	1.526E-03	4.768E-05	6.882E-06	5.563E-06	5.690E-06
	M.F.	1.001E + 05	1.309E + 01	1.616E + 00	4.888E-02	1.526E-03	6.485E-05	4.933E-05	**4.495E-05**	4.779E-05
	Std.	0.000E + 00	3.553E-15	0.000E + 00	1.388E-17	4.337E-19	3.010E-05	6.916E-05	2.908E-05	5.225E-05
F3	B.F.	3.000E + 04	6.122E + 02	3.122E + 01	2.867E-02	6.520E-05	5.921E-06	3.909E-07	7.990E-06	3.160E-06
	M.F.	3.000E + 04	7.755E + 02	6.660E + 01	5.085E + 00	4.436E-01	3.076E-01	5.889E-01	1.512E-01	**1.500E-01**
	Std.	0.000E + 00	3.265E + 02	4.219E + 01	1.769E + 01	1.088E + 00	1.055E + 00	1.226E + 00	3.090E-01	3.011E-01
F4	B.F.	3.028E + 08	6.047E + 04	3.940E-01	3.160E + 00	2.687E + 00	3.223E + 00	3.761E + 00	2.547E + 00	2.776E + 00
	M.F.	3.028E + 08	7.307E + 04	5.510E + 02	9.872E + 01	4.783E + 01	5.729E + 01	**3.213E + 01**	4.736E + 01	5.623E + 01
	Std.	0.000E + 00	1.837E + 04	4.405E + 02	1.445E + 02	1.097E + 02	1.104E + 02	6.362E + 01	9.613E + 01	1.061E + 02
F5	B.F.	1.997E + 01	1.430E + 01	3.786E + 00	1.766E-01	3.957E-03	1.221E-04	6.322E-05	1.899E-05	2.767E-05
	M.F.	1.997E + 01	1.430E + 01	3.786E + 00	3.098E-01	3.957E-03	4.169E-04	3.927E-04	**1.873E-04**	2.251E-04
	Std.	7.105E-15	1.685E-15	4.441E-16	5.806E-01	0.000E + 00	5.382E-04	3.805E-04	1.502E-04	1.620E-04
F6	B.F.	4.510E + 02	1.009E + 01	1.421E + 00	1.123E-01	2.308E-02	7.399E-03	1.018E-02	1.233E-02	9.926E-03
	M.F.	4.510E + 02	1.009E + 01	1.421E + 00	1.717E-01	9.270E-02	6.358E-02	7.841E-02	8.719E-02	**6.101E-02**
	Std.	1.705E-13	1.049E-14	4.441E-16	4.163E-02	8.413E-02	3.708E-02	4.623E-02	4.189E-02	5.152E-02

Significant values are in bold.

**Table A3:** Results according to change of nM ﻿(bold values indicate the best results for mean fitness).

Function	Contents	nM					
		1	2	4	6	8	10
F1	B.F.	5.386E-03	2.191E-04	2.083E-06	7.731E-07	4.547E-08	4.547E-08
	M.F.	4.094E-01	5.371E-03	1.247E-04	4.611E-05	3.349E-06	**3.183E-07**
	Std.	3.597E-01	5.372E-03	1.440E-04	1.341E-04	4.062E-06	2.900E-07
F2	B.F.	3.510E-02	1.097E-03	2.956E-04	4.768E-05	4.768E-05	4.768E-05
	M.F.	8.002E-02	1.259E-02	1.053E-03	3.052E-04	1.545E-04	**1.173E-04**
	Std.	3.325E-02	7.451E-03	6.028E-04	2.351E-04	1.033E-04	1.346E-04
F3	B.F.	2.536E + 00	9.162E-02	1.225E-03	3.175E-05	5.791E-05	3.774E-06
	M.F.	2.482E + 02	6.411E + 01	2.411E + 01	3.318E + 01	**2.111E + 01**	2.242E + 01
	Std.	3.860E + 02	1.307E + 02	6.947E + 01	8.905E + 01	6.902E + 01	6.895E + 01
F4	B.F.	2.961E + 00	2.387E + 00	2.744E + 00	2.268E + 00	3.101E + 00	2.759E + 00
	M.F.	2.326E + 02	1.018E + 02	4.824E + 01	3.823E + 01	4.298E + 01	**1.591E + 01**
	Std.	2.805E + 02	1.820E + 02	1.208E + 02	1.006E + 02	1.106E + 02	5.099E + 01
F5	B.F.	9.788E-02	5.258E-03	7.524E-04	2.504E-04	1.221E-04	1.221E-04
	M.F.	1.478E + 00	5.719E-01	1.231E-01	1.429E-01	1.296E-03	**8.620E-04**
	Std.	9.628E-01	9.632E-01	5.036E-01	6.133E-01	1.515E-03	1.521E-03
F6	B.F.	1.173E-01	6.194E-02	2.857E-02	2.435E-03	2.102E-02	2.766E-02
	M.F.	4.896E-01	1.957E-01	1.516E-01	1.296E-01	**1.109E-01**	1.113E-01
	Std.	2.627E-01	8.919E-02	1.169E-01	8.618E-02	7.406E-02	7.928E-02

Significant values are in bold.

**Table A4:** Results according to change of $\:{\upepsilon\:}$ ﻿(bold values indicate the best results for mean fitness).

Function	Contents	ε								
		0.00	0.02	0.05	0.07	0.10	0.15	0.20	0.30	0.40
F1	B.F.	4.547E-08	4.547E-08	4.547E-08	4.547E-08	4.547E-08	1.937E-06	1.180E-04	3.721E-02	6.012E-01
	M.F.	**4.547E-08**	**4.547E-08**	**4.547E-08**	**4.547E-08**	2.201E-07	9.973E-05	5.150E-03	5.749E-01	6.925E + 00
	Std.	1.323E-23	1.323E-23	1.323E-23	1.323E-23	2.439E-07	1.132E-04	4.983E-03	4.811E-01	5.263E + 00
F2	B.F.	4.768E-05	4.768E-05	4.768E-05	4.768E-05	4.768E-05	6.390E-04	5.503E-03	3.484E-02	8.525E-02
	M.F.	**4.768E-05**	**4.768E-05**	**4.768E-05**	**4.768E-05**	6.390E-05	1.484E-03	1.315E-02	1.045E-01	3.303E-01
	Std.	0.000E + 00	0.000E + 00	0.000E + 00	0.000E + 00	1.933E-05	7.289E-04	6.147E-03	4.766E-02	1.407E-01
F3	B.F.	1.197E-03	1.298E-05	6.366E-08	6.366E-08	7.602E-05	3.531E-04	2.503E-02	1.173E-01	6.702E + 00
	M.F.	2.443E + 02	7.075E + 01	9.765E + 01	5.410E + 00	**1.285E-01**	2.323E-01	1.714E + 00	3.186E + 00	2.073E + 01
	Std.	2.417E + 02	1.081E + 02	2.747E + 02	1.725E + 01	2.848E-01	5.346E-01	4.772E + 00	3.629E + 00	1.199E + 01
F4	B.F.	3.167E + 00	2.105E + 00	1.921E-01	3.108E + 00	2.232E + 00	8.267E-01	3.592E + 00	3.776E + 00	1.472E + 01
	M.F.	3.566E + 02	2.052E + 02	8.699E + 01	9.871E + 01	5.918E + 01	**1.388E + 01**	5.642E + 01	4.654E + 01	1.452E + 02
	Std.	5.676E + 02	4.507E + 02	1.356E + 02	1.527E + 02	1.184E + 02	3.709E + 01	1.096E + 02	9.775E + 01	1.555E + 02
F5	B.F.	1.221E-04	1.221E-04	1.221E-04	1.221E-04	1.221E-04	2.247E-03	1.781E-02	2.282E-01	9.831E-01
	M.F.	8.612E-01	3.648E-01	**1.221E-04**	5.150E-03	2.920E-04	7.471E-03	5.683E-02	7.677E-01	2.983E + 00
	Std.	1.181E + 00	8.703E-01	0.000E + 00	2.192E-02	1.995E-04	3.783E-03	4.749E-02	3.893E-01	6.095E-01
F6	B.F.	2.958E-02	1.725E-02	1.500E-02	1.242E-02	6.368E-07	2.540E-02	3.233E-02	2.305E-01	4.622E-01
	M.F.	3.519E-01	1.576E-01	9.901E-02	7.922E-02	**7.130E-02**	1.166E-01	1.299E-01	4.664E-01	7.395E-01
	Std.	2.975E-01	7.573E-02	6.132E-02	4.620E-02	5.568E-02	9.573E-02	7.923E-02	1.460E-01	2.034E-01

Significant values are in bold.

**Table A5:** Results according to change of $\:{\theta\:}_{R}$ ﻿(bold values indicate the best results for mean fitness).

Function	Contents	$\:{\theta\:}_{R}$							
		0.00	0.05	0.10	0.15	0.20	0.30	0.40	0.50
F1	B.F.	3.059E + 01	4.484E-06	7.540E-06	3.829E-06	1.936E-04	5.850E-02	5.911E-01	1.120E + 01
	M.F.	4.021E + 02	1.326E-04	2.814E-04	**1.176E-04**	1.694E-03	3.639E-01	3.833E + 00	3.956E + 01
	Std.	1.936E + 02	1.690E-04	5.455E-04	1.166E-04	1.980E-03	2.192E-01	2.781E + 00	2.230E + 01
F2	B.F.	5.989E-01	2.193E-04	4.292E-04	1.049E-04	1.707E-03	3.901E-02	9.501E-02	3.620E-01
	M.F.	3.148E + 00	**1.376E-03**	1.664E-03	1.494E-03	5.039E-03	9.674E-02	4.001E-01	9.982E-01
	Std.	1.470E + 00	8.406E-04	1.179E-03	8.032E-04	2.623E-03	3.685E-02	2.124E-01	3.058E-01
F3	B.F.	1.567E + 01	1.271E-04	2.495E-05	8.491E-04	6.533E-03	1.230E-01	7.382E-01	3.965E + 00
	M.F.	3.789E + 02	**1.828E-01**	5.385E-01	9.140E-01	6.593E-01	7.165E + 00	1.510E + 01	7.336E + 01
	Std.	2.209E + 02	3.529E-01	1.299E + 00	1.755E + 00	1.184E + 00	1.835E + 01	1.623E + 01	7.226E + 01
F4	B.F.	2.822E + 03	2.244E + 00	3.114E + 00	1.270E + 00	2.409E + 00	5.397E + 00	4.306E + 00	8.452E + 00
	M.F.	1.555E + 04	5.289E + 01	4.997E + 01	**4.989E + 01**	5.443E + 01	5.800E + 01	1.285E + 02	7.457E + 02
	Std.	1.398E + 04	1.167E + 02	1.356E + 02	8.158E + 01	1.061E + 02	9.701E + 01	1.485E + 02	7.272E + 02
F5	B.F.	2.357E + 00	6.309E-04	1.303E-03	1.572E-03	1.009E-02	2.521E-01	7.805E-01	2.711E + 00
	M.F.	8.980E + 00	7.424E-03	**6.157E-03**	6.296E-03	2.704E-02	9.524E-01	2.341E + 00	4.606E + 00
	Std.	2.484E + 00	5.216E-03	4.390E-03	4.924E-03	1.907E-02	6.311E-01	6.993E-01	1.002E + 00
F6	B.F.	1.920E + 00	2.872E-02	1.910E-02	2.472E-02	3.034E-02	2.399E-01	3.709E-01	9.483E-01
	M.F.	4.138E + 00	1.113E-01	**1.017E-01**	1.247E-01	1.580E-01	4.260E-01	7.308E-01	1.471E + 00
	Std.	1.594E + 00	4.786E-02	5.979E-02	6.924E-02	8.508E-02	1.534E-01	2.464E-01	4.887E-01

Significant values are in bold.
